# Influence of arteries, vein and placenta interactions on the blood flow in the human umbilical cord

**DOI:** 10.1371/journal.pone.0358954

**Published:** 2026-09-24

**Authors:** Hatim Machrafi, Xavier Capelle, Sébastien Grandfils, Christine Vanlinthout, Frédéric Kridelka, Pierre C. Dauby

**Affiliations:** 1 Institute of Materials Research (IUMAT), Hasselt University, Hasselt, Belgium; 2 Department of Obstetrics and Gynaecology, CHU of Liège, Liège, Belgium; 3 GIGA–In Silico Medicine, University of Liège, Liège, Belgium; Children’s Hospital of Orange County, UNITED STATES OF AMERICA

## Abstract

The mechanisms governing umbilical venous return in early pregnancy remain incompletely understood, particularly the roles and interactions of arterial pulsatility, umbilical cord mechanics, and placental compliance. In this study, we combine numerical modelling and *in vivo* measurements to investigate hemodynamic and mechanical interactions within the feto-placental circulation. A fully coupled three-dimensional fluid–structure interaction model of the umbilical cord—comprising two pulsatile arteries, a compliant vein, and Wharton’s jelly—was developed and connected to a zero-dimensional compliant placental model. The model incorporates arterial pressure pulsations, vessel wall deformation, and mechanical coupling within the closed arteries–placenta–vein system. In parallel, Doppler ultrasound measurements were performed in singleton pregnancies to assess umbilical venous flow at the placental insertion and in a free loop of the cord. Numerical results show that venous flow is dynamically modulated by arterial pulsations transmitted through both the cord and placenta, producing phase-shifted venous deformation and low-amplitude oscillations. Despite strong placental damping, the model predicts measurable venous flow oscillations with increasing amplitude toward the fetus. This behaviour depends on cord elasticity and placental compliance. The numerical model also points out that mechanical interactions between arteries, vein, and Wharton’s jelly contribute positively to the mean flow rate, demonstrating a pulsometer-like effect. Doppler measurements confirm that venous flow is not strictly steady and that oscillation amplitude increases toward the fetus, which is consistent with simulations. Overall, our combined computational and *in vivo* approach reveals that the interplay between arterial pulsations and placental compliance is a key driver of umbilical venous return. Finally, beyond their physiological significance, our findings may contribute to the design and optimization of future artificial placenta and extracorporeal fetal support systems.

## 1 Introduction

An adequate blood circulation through the umbilical cord between the fetus and the placenta is essential for the fetal development that needs proper gas and nutrient exchanges. In humans, two umbilical arteries carry blood from the fetus, poor in oxygen and rich in carbon dioxide, to the placenta. The return of oxygen rich blood from the placenta to the fetus occurs through the umbilical vein, which is surrounded by the two arteries. The possible mechanisms that allow sufficient venous return has long been questioned [1,2]. Indeed, one may question whether and how the role of the umbilical cord could be of significance. The umbilical arteries are quite closely wrapped around the vein. The overall structure, which also comprises Wharton’s jelly (WJ) with its mechanical properties, allows the cord to better resist compression, stretching and kinking during pregnancy and labour [3–6]. It has also been suggested that the structural characteristics of the cord are such that they could facilitate the venal blood return by means of the pulsating arteries acting a pulsating force on the vein, *i.e.,* a sort of pulsometer pump, rather than a peristaltic pump [2,7]. This work will investigate this effect numerically, but we will also analyse whether the placenta, which receives blood from the umbilical arteries and returns it to the vein, could also have some influence on the interactions between the arteries and the vein in the cord. To the best of our knowledge, these effects have not been studied in the context of a closed arteries-placenta-vein system with full fluid-structure interaction. Our work will describe the system numerically by developing a 3-D model of the cord, coupled with a simple 0-D model of the placenta with the objective to contribute to the understanding of the placenta-fetal blood flow behaviour.

The development of artificial placenta systems has renewed interest in the mechanisms governing fetoplacental hemodynamics. Experimental studies have demonstrated the sensitivity of fetal cardiovascular function to alterations in extracorporeal circuit resistance and flow conditions, emphasizing the importance of preserving physiological umbilical circulation [8–11]. However, the respective contributions of arterial pulsatility, umbilical cord mechanical properties, and placental compliance to venous return remain poorly understood. Elucidating these interactions may improve our understanding of normal fetoplacental physiology and contribute to the optimization of future extracorporeal fetal support technologies.

Quite some models have analysed the blood flow and the mechanical properties in an umbilical cord. Some consider the fluid flow to study the heat exchange from the fetus to its surroundings [12], but do not take into account the mechanical interactions between the arteries and the fetus, nor with the placenta. A specific analysis of the effect of several artery-related parameters (such as coiling, diameter, length) on the blood flow from an umbilical artery shows that above or below a certain threshold, these parameters are incompatible with fetal life [13]. However, in these studies no mechanical coupling is considered. In [14], the study considers the mechanics of the arteries expanding due to blood pressure oscillations, exerting pressure on the vein via Wharton’s jelly, which is considered as a spongy structure. The authors argue, by comparing 2-D and 3-D models to measured stresses, that this exerted pressure is at the origin of the venal blood return. However, the proposed study does not consider the role the placenta may play in this venal blood return. More work has been done in measuring and modelling the material properties of the umbilical cord [15,16]. Another work [17] uses a 1-D model to study the interactions between the umbilical arteries and the placenta and their influence on the flow velocity and terminal resistance, but the system is not connected to a vein. A more detailed model on placental blood flow is presented in [18], while fetal circulation connected to an artificial 0-D placenta is described in [19]. Note that a 0-D placenta is also considered in [20] but in the study of maternal circulation.

In the present work, we consider a fully coupled model of the interaction between the blood flow in the arteries and that in the vein. This interaction happens in two ways. The first is a mechanical one, where the blood pressure in the arteries exerts a force on their walls. This force or pressure is transmitted through Wharton’s jelly to the vein’s wall, influencing thereby the venous blood flow. Of course, the interaction also goes back towards the arteries, even if the effect seems to be of negligible importance. The second interaction mechanism between the flow in the arteries and in the vein relies on the influence of the compliant placenta, that regulates the pressure drop and the flow between the arteries’ exiting blood flow and the venous entering one. In the present paper, we intend to describe the influence of the placenta on the behaviour of the cord, but we are not interested in a detailed analysis of the physical phenomena taking place inside that organ. For that reason, a 0-D (or lumped-parameter) model of the placenta is considered in our numerical model.

To explore whether the numerically described interactions have measurable physiological correlates in humans, we next investigate umbilical venous flow in vivo using Doppler velocimetry. Clinically, both hypo-coiled and hyper-coiled umbilical cords have been shown to be significantly associated with an increased prevalence of adverse fetal outcomes, including preterm birth, fetal distress, small-for-gestational-age neonates, congenital anomalies, fetal growth restriction, heart rate abnormalities, and fetal demise [21–23]. Several studies have also demonstrated that the degree of umbilical arterial coiling and the morphological characteristics of the umbilical cord influence fetal hemodynamic parameters, as assessed by Doppler ultrasound [24–27]. However, despite the experimental work by Reynolds [1] in the ovine model, to our knowledge, no study has specifically investigated, in the human fetus, the impact of arterial pulsatility on umbilical venous blood flow. This gap in knowledge provides the rationale for the experimental part of the present study, which aims to explore, using Doppler velocimetry, the effects of umbilical arterial pulsations –modulated by arterial coiling and Wharton’s jelly– on umbilical venous flow dynamics in the human feto-placental circulation.

The next section presents our *Methods*, both for the numerical approach and for the experimental measurements. Section 3 describes our main *Results*, which are then analysed in detail in the subsequent *Discussion* section. The limitations of our approach are also carefully presented. Finaly, we draw the *Conclusion* and present several possible extensions and applications of our work.

## 2 Methods

### 2.1 Numerical model

**Fig 1:**
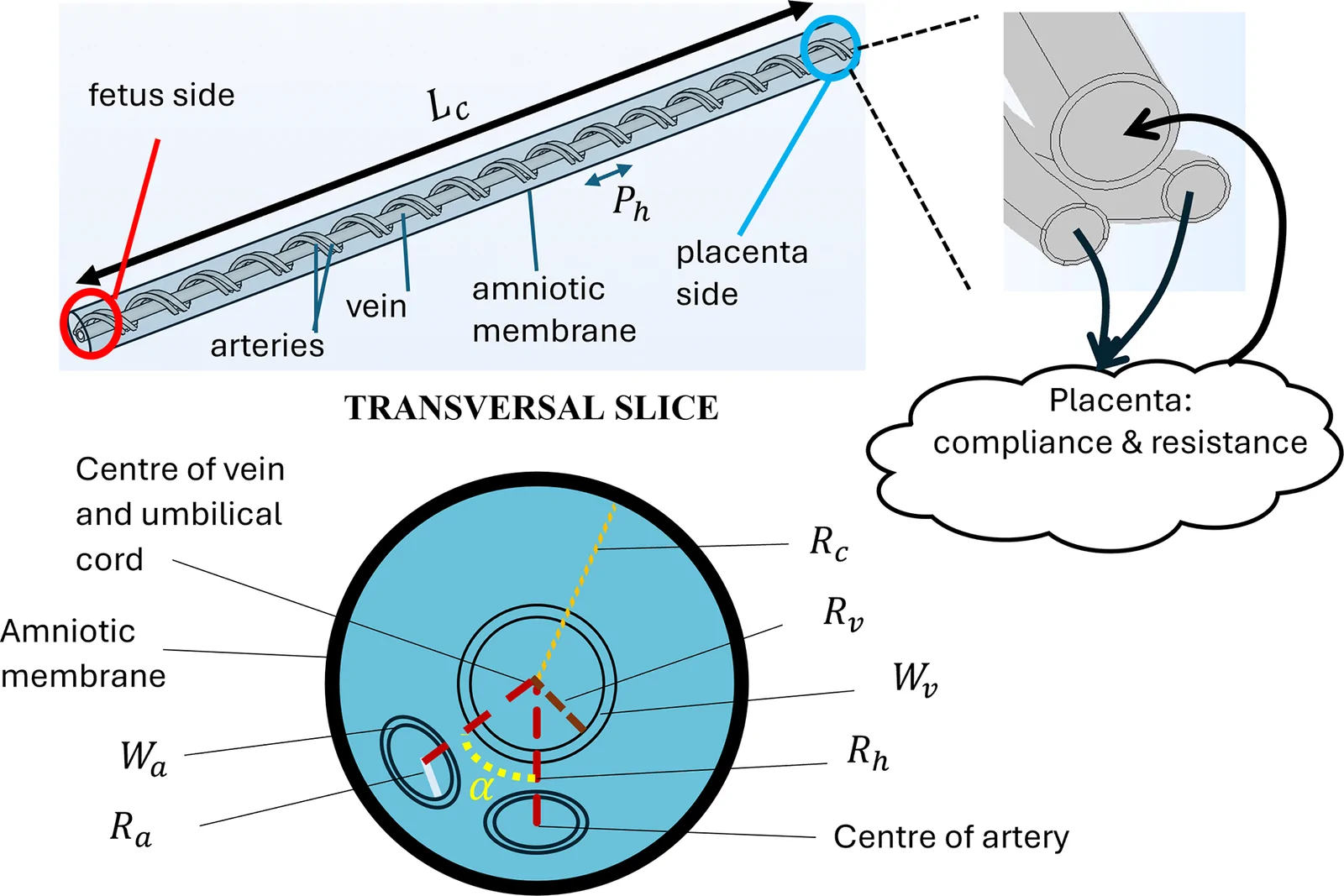
Schematic representation of the modelled umbilical cord with placenta connection. The geometry of the system is defined in a reference configuration for which the whole system is assumed to be submitted to a constant reference pressure $p_{\text{0}}$. In these conditions, the outer shell of the umbilical cord is of circular cylindrical shape with length $L_{c}$ and radius $R_{c}$. The vein is a circular cylinder, with inner radius $R_{v}$ and wall thickness $W_{v}$; its axis is at the centre of the cord. Each artery is a tube whose centre line is a circular helix with a pitch $P_{h}$ and a radius $R_{h}$. The section of the arteries in planes normal to the helix are circles of inner radius $R_{a}$ and the thickness of the wall is $W_{a}$ (sections of arteries in transversal slices look elliptical) and the angle between the two arteries is α.

The umbilical cord, with its connections to the fetus and the mother is a complex system, whose modelling requires simplifying assumptions to keep the model and system of equations tractable. Our assumptions will be discussed in Section 4.4 below and we present here only the resulting description. The umbilical cord, schematized in Fig 1, is modelled by a 3-D geometry, comprising two helix-shaped arteries winded around a cylindrical tube that stands for the vein, and the so-called WJ that fills the space between the vessels and the external surface of the cord, which is assumed to be a fixed, rigid and circular cylinder.

The dimensions of the system which are introduced in Fig 1 are defined in a *reference configuration,* for which the whole system is assumed to be submitted to a constant reference pressure $p_{\text{0}}$, the latter being the minimum arterial pressure at the entrance of the umbilical cord. WJ and the arteries and vein walls are considered as linear isotropic elastic materials, with constant material properties. The fetus blood in the vessels is assumed to be Newtonian, with a constant dynamic viscosity $\mu_{b}$, and incompressible, with density $\rho_{b}$. With the above assumptions, the equations that govern the (liquid) blood are given by [28–30]:


$$\begin{array}{c} \begin{array}{c} \nabla \cdot {\overset{⇀}{v}}_{b}=\text{0}, \end{array} \end{array}$$ (1)

$$\begin{array}{c} \begin{array}{c} \rho_{b}\frac{\partial {\overset{⇀}{v}}_{b}}{\partial t}=-\rho_{b}\left({\overset{⇀}{v}}_{b}\cdot \nabla \right){\overset{⇀}{v}}_{b}-\nabla p_{b}+\mu_{b}\nabla^{\text{2}}{\overset{⇀}{v}}_{b}, \end{array} \end{array}$$ (2)

where ${\overset{⇀}{v}}_{b}$ and $p_{b}$ are the barycentric velocity and pressure fields, respectively, whereas t is the time variable. Equations (1) and (2) stand for the continuity and momentum equations. After passing through the arteries, and before entering the vein, the blood goes into the placenta, which is described by a 0-D (or lumped parameter) model of a compliant cavity of compliance $C_{p},$ followed by a hydraulic resistance $R_{p}$ [19]. The equations describing the placenta are then given by:


$$\begin{array}{c} \begin{array}{c} C_{p}\frac{dp_{p}(t)}{dt}=Q_{a}(t)-Q_{v}(t), \end{array} \end{array}$$ (3)

$$\begin{array}{c} \begin{array}{c} Q_{v}(t)=\frac{p_{p}(t)-p_{v}(t)}{R_{p}}, \end{array} \end{array}$$ (4)

where $p_{p}$ is the pressure in the placenta, $p_{v}$ the inlet pressure of the vein (which is assumed to be spatially uniform), while $Q_{a}(t)$ and $Q_{v}(t)$ are, respectively, the (total) flow rates coming into the placenta from the arteries and going into the vein from the placenta, both depending on time.

The equation that governs the solid phases (WJ and walls of vein and arteries) is given by [28]:


$$\begin{array}{c} \begin{array}{c} \rho \;\frac{\partial^{\text{2}}\overset{⇀}{s}}{\partial t^{\text{2}}}=\nabla \cdot \sigma_{s}, \end{array} \end{array}$$ (5)

where $\overset{⇀}{s}$ is the displacement field, ρ the material density, and $\sigma_{s}$ the stress tensor in the solid phase. As we assume Hookean behaviour, the stress tensor is given by Hooke’s law, with


$$\begin{array}{c} \begin{array}{c} \sigma_{s}=\lambda_{L}tr\left(\varepsilon \right)I+\text{2}\mu_{L}\varepsilon , \end{array} \end{array}$$ (6)

where I is the identity tensor and tr(ε) is the trace of the strain deformation tensor, ε**,** where the latter is given by


$$\begin{array}{c} \begin{array}{c} \varepsilon =\frac{\text{1}}{\text{2}}\left({\left(\nabla \overset{⇀}{s}\;\right)}^{T}+\nabla \overset{⇀}{s}\;\right). \end{array} \end{array}$$ (7)

In (6), $\lambda_{L}$ and $\mu_{L}$ are the Lamé coefficients, which can also be expressed in terms of the scalar Young modulus E and the Poisson’s ratio ν ($\lambda_{L}=E\nu (\text{1}+\nu {)}^{-\text{1}}(\text{1}-\text{2}\nu {)}^{-\text{1}}$ and $\mu_{L}=\frac{E(\text{1}+\nu {)}^{-\text{1}}}{\text{2}}$).

Let us now describe the boundary conditions that must be introduced between the different parts of our system. First at the inlet of the arteries, we impose a (spatially uniform) sinusoidal pressure variation, describing the given pressure of the blood coming from the fetus’ heart and a velocity field normal to the surface:


$$\begin{array}{c} \begin{array}{c} p_{b}=p_{\text{0}}+\frac{\Delta p}{\text{2}}\;\left[\text{1}+\text{sin}(\text{2}\pi ft)\right]\text{ and }\overset{⇀}{v}\cdot \overset{⇀}{t}=\text{0}, \end{array} \end{array}$$ (8)

where Δp/2 is the amplitude of the pressure oscillations, $p_{\text{0}}$ is the minimum pressure and $\overset{⇀}{t}$ represents any unit vector tangent to the inlet surface. Note that the minimum pressure $p_{\text{0}}$ is the pressure that has been introduced in Fig 1 to describe the reference geometry of our system. The connection between the arteries and the placenta is modelized by assuming a spatially uniform pressure $p_{b}$ at the exit of the arteries, this pressure being equal to the placenta pressure $p_{p}$:


$$\begin{array}{c} \begin{array}{c} p_{b}=p_{p}. \end{array} \end{array}$$ (9)

At the entrance of the vein, the blood pressure $p_{b}$ is equal to the “inlet vein pressure” $p_{v}$ that appears in eq. (4):


$$\begin{array}{c} \begin{array}{c} p_{b}=p_{v}. \end{array} \end{array}$$ (10)

Note that we also assume that the blood velocity profile at the vein inlet is spatially uniform (flat profile, assumed to be a consequence of multiple capillaries “pouring” into the vein, hindering developed flow). Finally, at the outlet of the vein, we assume:


$$\begin{array}{c} \begin{array}{c} p_{b}=p_{out}\text{ and }\overset{⇀}{v}\cdot \overset{⇀}{t}=\text{0}, \end{array} \end{array}$$ (11)

where $p_{out}$ is an assumed constant and given pressure when blood arrives in the fetus. The boundary conditions between the solid parts of the system and between the vessels’ walls and the blood express the continuity of stress and displacement. These conditions write:


$$\begin{array}{c} \begin{array}{c} {\overset{⇀}{v}}_{I}={\overset{⇀}{v}}_{II}\text{ and }\sigma_{I}\cdot \overset{⇀}{n}=\sigma_{II}\cdot \overset{⇀}{n}, \end{array} \end{array}$$ (12)

where I and II represent the two sides of the different boundaries (blood and inner wall of a vessel, or outer wall of a vessel and WJ),$\;\overset{⇀}{v}$ is the velocity vector, σ is the stress tensor and $\overset{⇀}{n}$ is the normal vector to these surfaces. In the solid parts, the stress tensor ${\sigma =\sigma }_{s}$ is given by (6), while in the blood, the stress tensor is written $\sigma_{b}=-\left(p_{l}-p_{\text{0}}\right)𝐈+\mu_{l}\left({\left(\nabla \overset{⇀}{v}\right)}^{T}+\nabla \overset{⇀}{v}\right)$, with the pressure contribution measured with respect to the constant pressure $p_{\text{0}}$ introduced in the definition of the reference configuration. In the two limiting transversal planes of the cord, the axial displacements of the vein and arteries walls, and of WJ are zero, while radial deformations are not constrained, which can be mathematically expressed as:


$$\begin{array}{c} \begin{array}{c} \overset{⇀}{v}\cdot \overset{⇀}{n}=\text{0}\text{ and }\overset{⇀}{t}\cdot \sigma_{s}\cdot \overset{⇀}{n}=\text{0}. \end{array} \end{array}$$ (13)

Finally, the outer surface of the umbilical cord is assumed to be fixed and non-deformable, which corresponds to the following condition for WJ:


$$\begin{array}{c} \begin{array}{c} \overset{⇀}{s}=\text{0}. \end{array} \end{array}$$ (14)

The above model contains several parameters, whose values must be specified. In our work, we will consider the values associated to the human umbilical cord at about 20–22 weeks of gestation, but it worth mentioning right away that not all values are precisely known and available in the literature. First, the minimum pressure at the arteries’ entrance, which is also the reference pressure needed to define the geometry, is chosen as $p_{\text{0}}=\text{2000 Pa}$ [31]. Consider now the geometrical parameters defined at pressure $p_{\text{0}}$. The length and diameter of the cord are chosen as $L_{c}=\text{4}\text{0}\text{ cm}$ [32] and $R_{c}=\text{8}\text{ mm}$ [33], while the dimensions of the vessels are given by $R_{a}=\text{1}.\text{3}\text{ mm}$, $R_{v}=\text{2}.\text{4}\text{ mm}$ [33], $W_{a}=\text{0}.\text{2}\text{ mm}$, $W_{v}=\text{0}.\text{4}\text{ mm}$ (the wall thicknesses are taken a bit lower than those at birth [34], in lack of better alternatives). The pitch and radius of the helix are $P_{h}=\text{2}.\text{5}\text{ cm}$ [14] and a radius $R_{h}=\text{5}\text{ mm}$ (value estimated in consideration of the other geometric data, keeping the arteries approximately equidistant between the vein and the edge of the umbilical cord), while the angular gap α between the two helical arteries is 65° (estimation from a picture at birth [35], in lack of better alternatives). Let us now consider the different material properties. In [36], blood density is reported to be slightly higher than that of water, but another work [37] conflicted this stating it to be nearly the same. So, we took $\rho_{b}=\text{9}\text{9}\text{4}\text{ kg}{\text{ m}}^{-\text{3}}$, which corresponds to water at the body temperature. For blood viscosity, we took $\mu_{b}=\text{2}\times {\text{1}\text{0}}^{-\text{3}}\text{ Pa s}$, which is the average between the viscosity given in [36] and that corresponding to high shear [38]. The heartbeat frequency is chosen as $f=\text{2}.\text{5}\;{\text{s}}^{-\text{1}}$ (period equal to 0.4 s), while the minimum and maximum pressure at the arteries entrance are $p_{\text{0}}=\text{2000 Pa}$ and $p_{\text{0}}+\Delta \text{p}=\text{6000 Pa}$ [31]. The pressure at the vein exit is $p_{out}=\text{7}\text{0}\text{0}\text{ Pa}$ [39]. The density of WJ was obtained from [40]. The value $\rho_{WJ}=\text{1}\text{6}\text{3}\text{0}\text{ kg}{\text{ m}}^{-\text{3}}\;$is rather large and probably a bit questionable, but an order of magnitude analysis of the terms of eq. (5) shows that this should not influence the results. WJ Young’s modulus $E_{WJ}=\text{2}\text{ kPa}$ was derived from [41,42] considering low strain, as is the case in our study. For WJ Poisson ratio, we took $\nu_{WJ}$=0.4, which is inside the values reported in [41] and allows avoiding numerical convergence problem when WJ exhibits near-incompressible behaviour. The density $\rho_{a}=\text{1}\text{3}\text{0}\text{0}\text{ kg }{\text{m}}^{-\text{3}}$ of the arteries’ wall is taken from [43], while that for the vein wall $\rho_{v}=\text{1}\text{2}\text{0}\text{0}\text{ kg }{\text{m}}^{-\text{3}}\;$is taken from [36]. In [43], a study on stenosed arteries reports a value of 910 kPa for the Young’s modulus. As stenosed arteries have increased Young’s modulus [44], we took the smaller value $E_{a}=$500 kPa. For the vein, many values can be found in the literature, with significant discrepancies among studies [45–47]. Since we are interested in a value corresponding to low strain, we took $E_{v}$=5 kPa which is reported in [48]. The Poisson ratio of the artery walls is reported in [43,49], and we took the value of $\nu_{a}$=0.499, while for the vein we took $\nu_{v}$=0.4 [50]. Finally, first estimates for the placenta resistance and compliance were obtained from [19], and then modified and fitted heuristically to obtain appropriate blood flows as provided in [51]. The values we took are $C_{p}=\text{4}\times {\text{10}}^{-\text{9}}\;{\text{m}}^{\text{3}}\text{P}{\text{a}}^{-\text{1}}$ and $R_{p}={\text{10}}^{\text{9}}\text{ Pa s }{\text{m}}^{-\text{3}}$. The above values of the parameters define what we call the *reference case* and are gathered in Table 1, which also provides the nomenclature of our model.

**Table 1:** Working conditions and values of the parameters for the *reference case* and nomenclature associated to our model (subscripts b , p , a , v , WJ, s, I and II designate the blood, the placenta, the arteries, the vein, Wharton’s jelly, solid phase in general and adjacent boundaries I and II, respectively).

**Working Conditions**		
**Symbol [units]**	**Value**	**Nomenclature**
$p_{\text{0}}$ [Pa]	2000	Minimum pressure at the arteries’ entrance
Δp/2 [Pa]	2000	Pressure oscillation amplitude at the arteries’ entrance
$p_{out}$ [Pa]	700	Vein exit pressure
f [s^-1^]	2.5	Heartbeat frequency
**Material properties**		
**Symbol [units]**	**Value**	**Nomenclature**
$C_{p}$ [m^3^/Pa]	4×10^−9^	Placenta compliance
$R_{p}$ [Pa s/m^3^]	1×10^9^	Placenta resistance
$E_{a}$ [Pa]	5×10^5^	Artery wall Young’s modulus
$E_{v}$ [Pa]	5×10^3^	Vein wall Young’s modulus
$E_{WJ}$ [Pa]	2×10^3^	WJ Young’s modulus
$\nu_{a}$ [-]	0.499	Artery wall Poisson ratio
$\nu_{v}$ [-]	0.4	Vein wall Poisson ratio
$\nu_{WJ}$ [-]	0.4	WJ Poisson ratio
$\rho_{a}$ [kg/m^3^]	1300	Density of arteries wall
$\rho_{v}$ [kg/m^3^]	1200	Density of vein wall
$\rho_{WJ}$ [kg/m^3^]	1630	Density of WJ
$\rho_{b}$ [kg/m^3^]	994	Blood density
$\mu_{b}$ [Pa s]	2×10^−3^	Blood dynamic viscosity
$L_{c}$ [m]	0.40	Length of the umbilical cord
$R_{c}$ [m]	8×10^−3^	Radius of the umbilical cord
$R_{h}$ [m]	5×10^−3^	Radius of the circular helices defining the arteries
$R_{a}$ [m]	1.3×10^−3^	Radius of the arteries
$R_{v}$ [m]	2.4×10^−3^	Radius of the vein
$W_{a}$ [m]	0.2×10^−3^	Wall thickness of the arteries
$W_{v}$ [m]	0.4×10^−3^	Wall thickness of the vein
$P_{h}$ [m]	25×10^−3^	Pitch of the circular helix
α [°]	65	Angle between two arteries
**Variables [units]**		
**Symbols [units]**		**Nomenclature**
${\overset{⇀}{v}}_{b}$ [m/s]		Blood velocity field
$p_{b}$ [Pa]		Blood pressure
t [s]		Time
Q [m^3^/s]		Flow rate
$\overset{⇀}{s}$ [m]		Displacement field
$\sigma_{i}$ [Pa]		Stress sensor of medium i=a, v, s, WJ, I, II
ε [-]		Strain deformation tensor

In addition to the reference case, we will also consider other situations, with different values of the parameters. More precisely, we will first analyse the effect of changing the stiffness of the umbilical cord, by increasing the Young moduli of the arteries’ and vein’s walls and of WJ. Then we will also consider a decreased value for the placenta compliance. These two cases will provide a sort of sensitivity analysis of our model with respect to important parameters that shape the interactions between arteries and vein in the umbilical cord.

The system of equations (1)-(14), with the associated parameters, has been solved numerically and all the results presented below describe only the long-term behaviour of the system, *i.e.,* the asymptotic periodic behaviour induced by the periodic driving of eq. (8) and obtained after removing the transient evolution. The commercial software ComSol Multiphysics has been used to solve the equations, using an unsteady solver with a maximum time step of 0.01 s. The mesh velocities are calculated in accordance with the arbitrary Lagrangian-Eulerian (ALE) formulation. This ALE-based finite element method allows the computational mesh inside the domains to move arbitrarily to optimize the shapes of the elements as well as the mesh on the boundaries and interfaces to move along, tracking thereby quite accurately the position of the liquid-solid interfaces. The convergence condition we have used to define the asymptotic periodic behaviour is based on the value of the mean mass flow across the cord, whose first three digits must be constant from one to the next. Note that this criterium, which is already rather challenging as far as the calculation time is concerned (about two full days for 10 heart beats on a server with sixteen 32Gb cores), does not allow to reach a perfect precision for some quantities that change very little during a period (pressure variations close to the vein exit, for instance).

### 2.2 Experimental approach

Between 1^st^ of February 2021 and 30^th^ of September 2022, 88 pregnant women were prospectively enrolled at the University Hospital of Liège (CHU de Liège) and underwent a dedicated ultrasound examination between 11 and 14 weeks of gestation. The study was approved by the Ethics Committee of the University Hospital of Liège (Belgium) under EUDRA CT number B707-2020-0000-49. Written informed consent was obtained from all participants. Inclusion criteria were singleton pregnancy, absence of major fetal anomalies on ultrasound, absence of pregnancy-related pathology at the time of inclusion, and planned delivery at our institution. Twenty-six patients were excluded during follow-up due to poor ultrasound conditions, miscarriage, umbilical cord abnormalities, fetal anatomical anomalies, or delivery in another institution.

**Fig 2:**
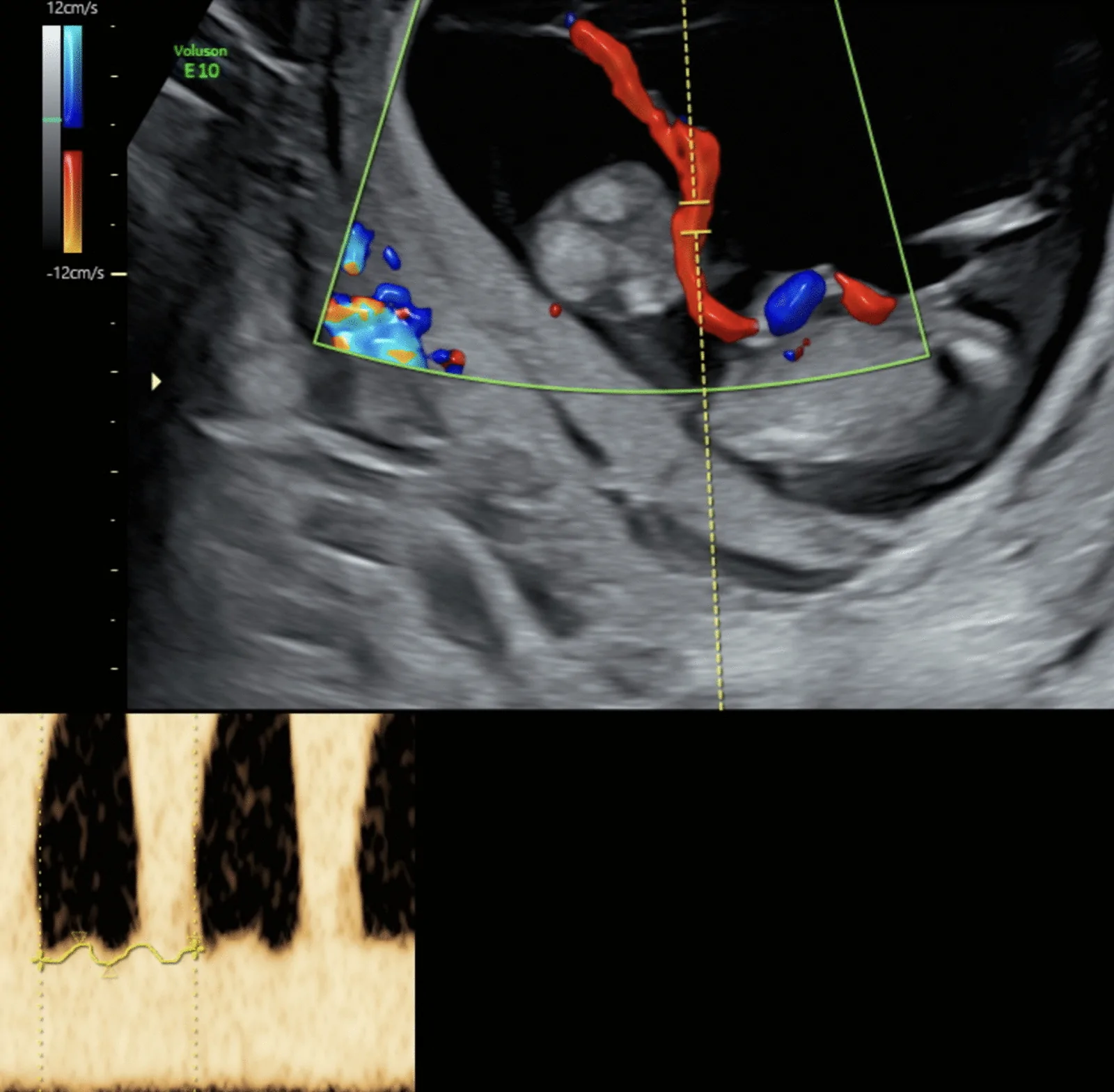
Ultrasound measurement of the maximum velocity in the umbilical vein at a free-floating loop in the middle part of the cord. Oscillations in the maximum velocity waveform are clearly visible (bottom left).

Initial ultrasound examinations were performed by a single senior operator (XC). Inter-observer agreement for umbilical vein blood flow velocity measurements was assessed by two additional operators (CVL and SG) using the intraclass correlation coefficient (ICC). All ultrasound examinations were performed using either a 4–8 MHz transabdominal transducer (Voluson E8) or a 2–8 MHz transabdominal or 5–9 MHz transvaginal transducer (Voluson E10), both equipped with multi-frequency transmission and tissue harmonic imaging capabilities (GE Healthcare). Gestational age was established based on crown–rump length (CRL) measurement [52]. The time evolution of the maximum blood flow velocity in the umbilical vein (see the yellow curve in the left lower part of Fig. 2) was assessed using pulsed-wave Doppler.

Two measurement sites were selected: a segment located 5–15 mm from the placental insertion of the cord, which is referred to by using a lower index *PL* (for placenta) in the notation, and a free-floating loop, with lower index *FL*, in the intermediate portion of the umbilical cord. Velocity measurements were obtained in straight, non-compressed segments of the vein, avoiding areas potentially influenced by fetal parts. An insonation angle of less than 20° between the Doppler beam and the axis of the umbilical vein was required. Angle correction was applied, when necessary [53,54]. The Doppler sample volume encompassed the entire diameter of the vein. Doppler recordings lasted at least 5 seconds, with a velocity scale set between 0 and 12 cm/s. Measurements were performed during fetal quiescence and maternal breath-holding (Fig 2). Umbilical vein diameters $D_{PL}$ and $D_{FL}$ were measured at both velocity sampling sites, from inner wall to inner wall, using a magnified image and with the ultrasound beam perpendicular to the vessel’s long axis. Each measurement was performed twice and averaged [55]. All sonographic data were digitally stored for retrospective analysis.

The flow in the umbilical vein, classically described as non-pulsatile under physiological conditions, exhibits oscillations in varying proportions during the first trimester. We observed reproducible oscillations in the time dependent maximum velocity waveform. Therefore, venous velocity variations were quantified, at both velocity sampling sites, by calculating the amplitude oscillation $\Delta V=V_{S}-V_{D}$, where $V_{S}$ and $V_{D}$ are the maximum (systolic) and minimum (diastolic) over a cardiac cycle of the maximum velocity. In addition, at both measurement sites, a time averaged umbilical venous velocity $V_{mean}=(\frac{\text{1}}{\text{2}})\left(V_{S}+V_{D}\right)$ was estimated by calculating the mean between the maximum and minimum velocities over the pulsatile cycle. Below, the results are presented in terms of mean and standard deviation (SD) for quantitative parameters. The change in velocity between sites was evaluated by the paired Student t-test and the results were significant at the 5% confidence level (p < 0.05). The reproducibility of the measurements was assessed by the intra-class correlation coefficient (ICC) and its 95% confidence interval (95% CI).

## 3 Results

### 3.1 Numerical results

**Fig 3:**
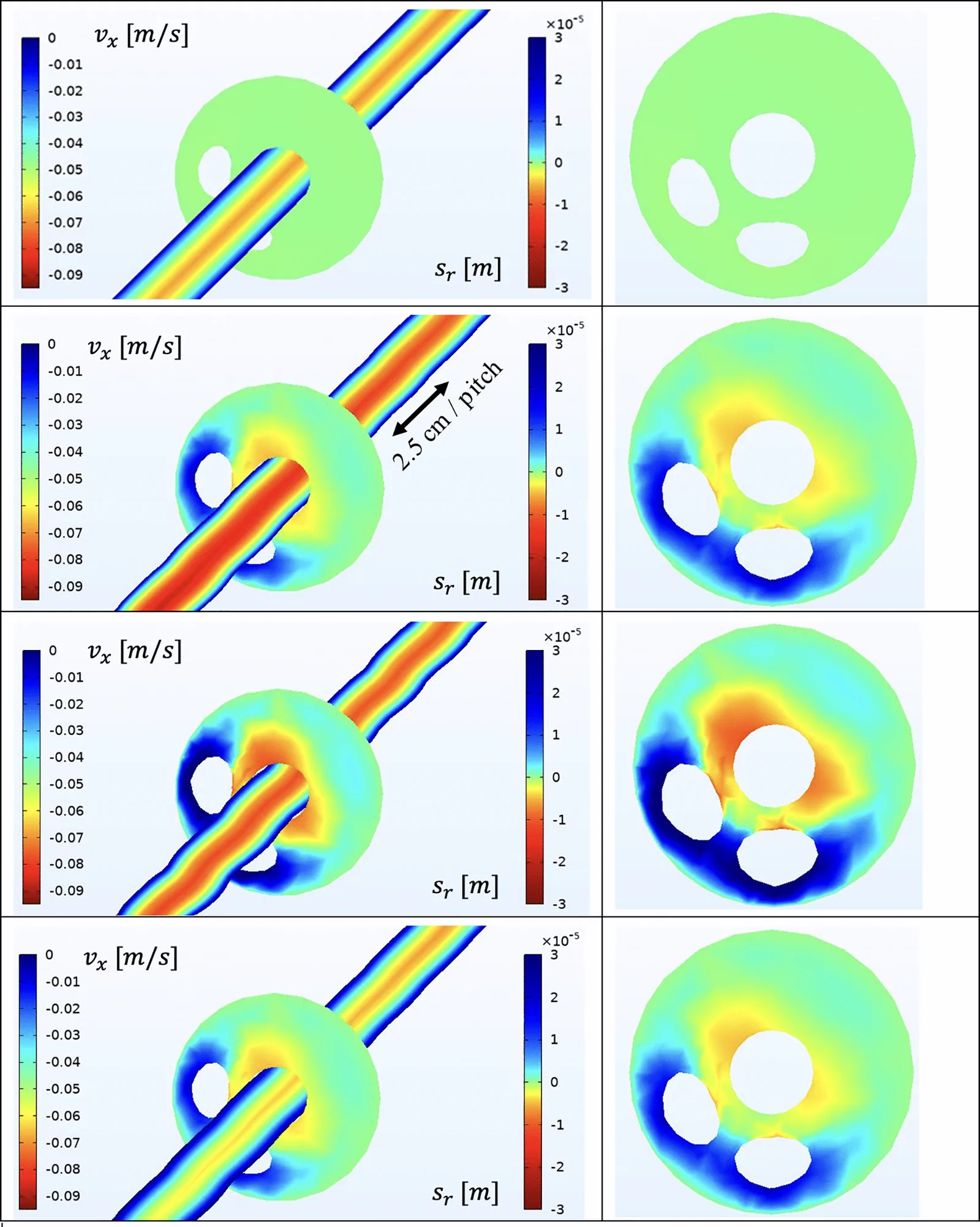
Four snapshots (at $t=t_{0}$, $t_{0}+0.1$, t= $t_{0}+0.2$ and $t_{0}+0.3$ s from top to bottom, respectively), where $t=t_{0}$ and t= $t_{0}+0.2$ correspond to minimum and maximum artery pressure, respectively. In the emphasized vertical slice in the four left images, which is at 10 cm from the vein’s exit, the radial Wharton jelly’s displacement, $s_{r}$, is represented. These vertical slices are presented in enlarged form in the four right images. The “diagonal” stripes in the left images show the axial component of the vein velocity, $v_{x}$. For illustration, the length scale of one artery pitch, i.e., 2.5 cm, is shown.

In this section, we present and discuss first the results corresponding to the parameters given in Table 1, which we will refer to as the *reference case*. As mentioned earlier, we also consider a stabilized oscillatory regime. Fig 3 provides a first general overview of the behaviour of the system for the reference case. Four snapshots are presented, which correspond to t= $t_{\text{0}}$, $t_{\text{0}}+\text{0}.\text{1}\text{ s}$, $t_{\text{0}}+\text{0}.\text{2}\text{ s}$ and $t_{\text{0}}+\text{0}.\text{3}\text{ s}$, where $t_{\text{0}}$ is an arbitrary time at which the arteries’ entrance pressure equals the minimum pressure $p_{\text{0}}$ (the four instants correspond to the minimum, average, maximum and again average artery pressures, respectively). The different plots describe the blood axial velocity field in a “diagonally pictured” axial slice of the vein and the radial displacement of WJ in a cross-section of the cord located at 10 cm from the vein exit.

When the pressure in the arteries has the minimum value ($p=p_{\text{0}}$ at $t=t_{\text{0}}$), the displacement in WJ is zero and the vein is not deformed. In contrast, when the pressure in the arteries reaches the maximum value ($p=p_{\text{0}}+\Delta p$ at $t=t_{\text{0}}+\text{0}.\text{2}\text{s}$), one clearly sees the deformation of the vein and the images in the third row of Fig 3 emphasize that the vein is no longer a straight cylinder but somewhat bent by the surrounding arteries and, as will appear later, slightly crushed. The numerical data from the simulations also show that the maximum velocity in the vein is then 7.94 cm/s.

**Fig 4:**
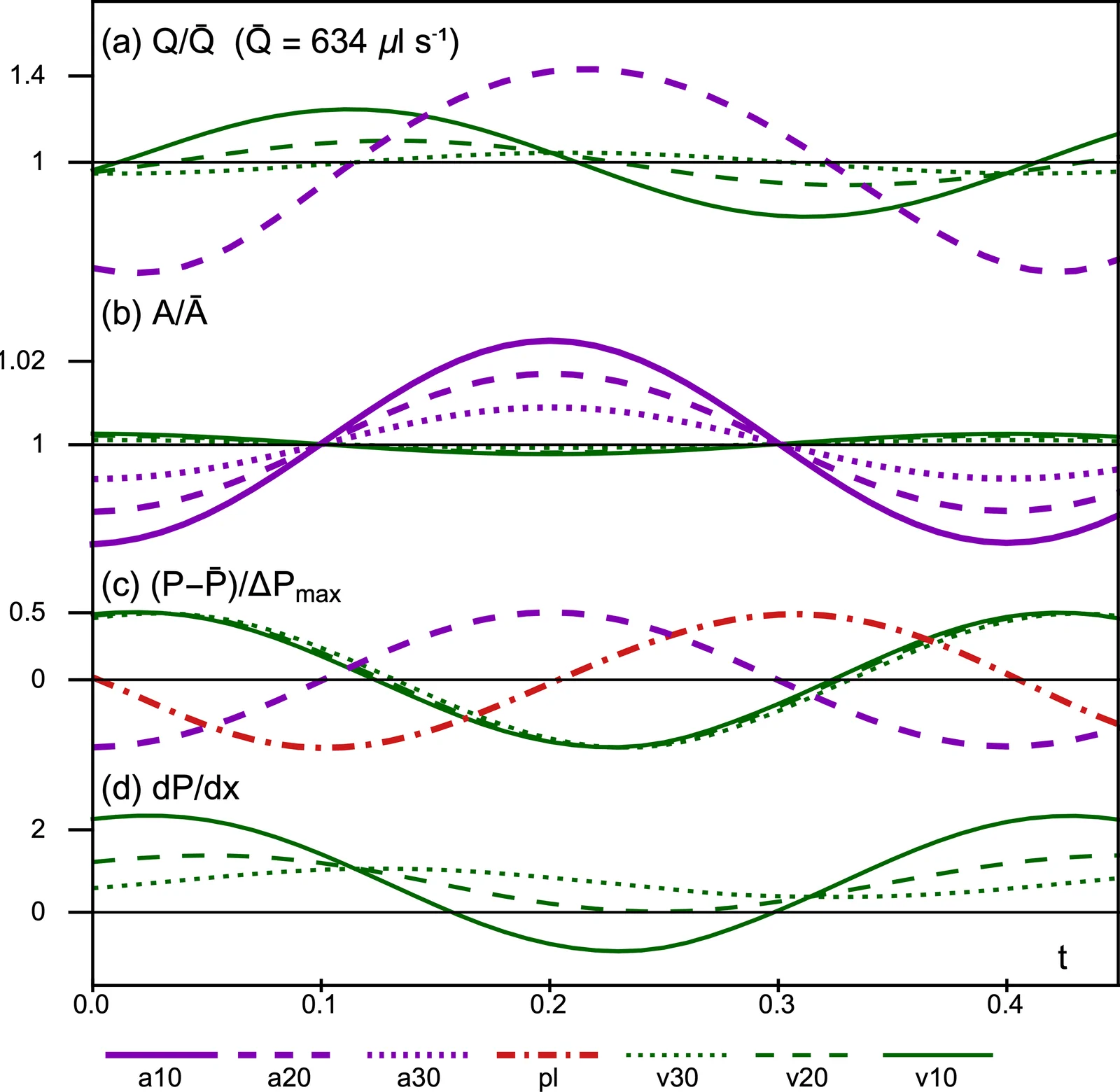
Time evolution of important quantities in the system for the reference case (parameters given in Table 1; plots slightly smoothened in order to remove irrelevant numerical noise). In the labels of the curves, *a*, *v* and *pl* refer to the arteries, the vein and the placenta respectively and the accompanying numbers (a30, or v10, for instance) indicate the position x along the cord, measured from the fetus and expressed in cm, for which the different quantities are plotted. (a) Relative flow rates $Q/\overset{―}{Q}$, where $\overset{―}{Q}$ is the time average of the flow across the cord. (b) Relative areas of the cross-sections $A/\overset{―}{A}$ for the two arteries and for the vein, with $\overset{―}{A}$ is the time average of these areas. (c) Pressures expressed in terms of the time averaged pressures $\overset{―}{P}$ and the amplitudes of the pressure variations $(\Delta P{)}_{max}$. (d) Pressure gradients (note that x is expressed in cm).

**Fig 5:**
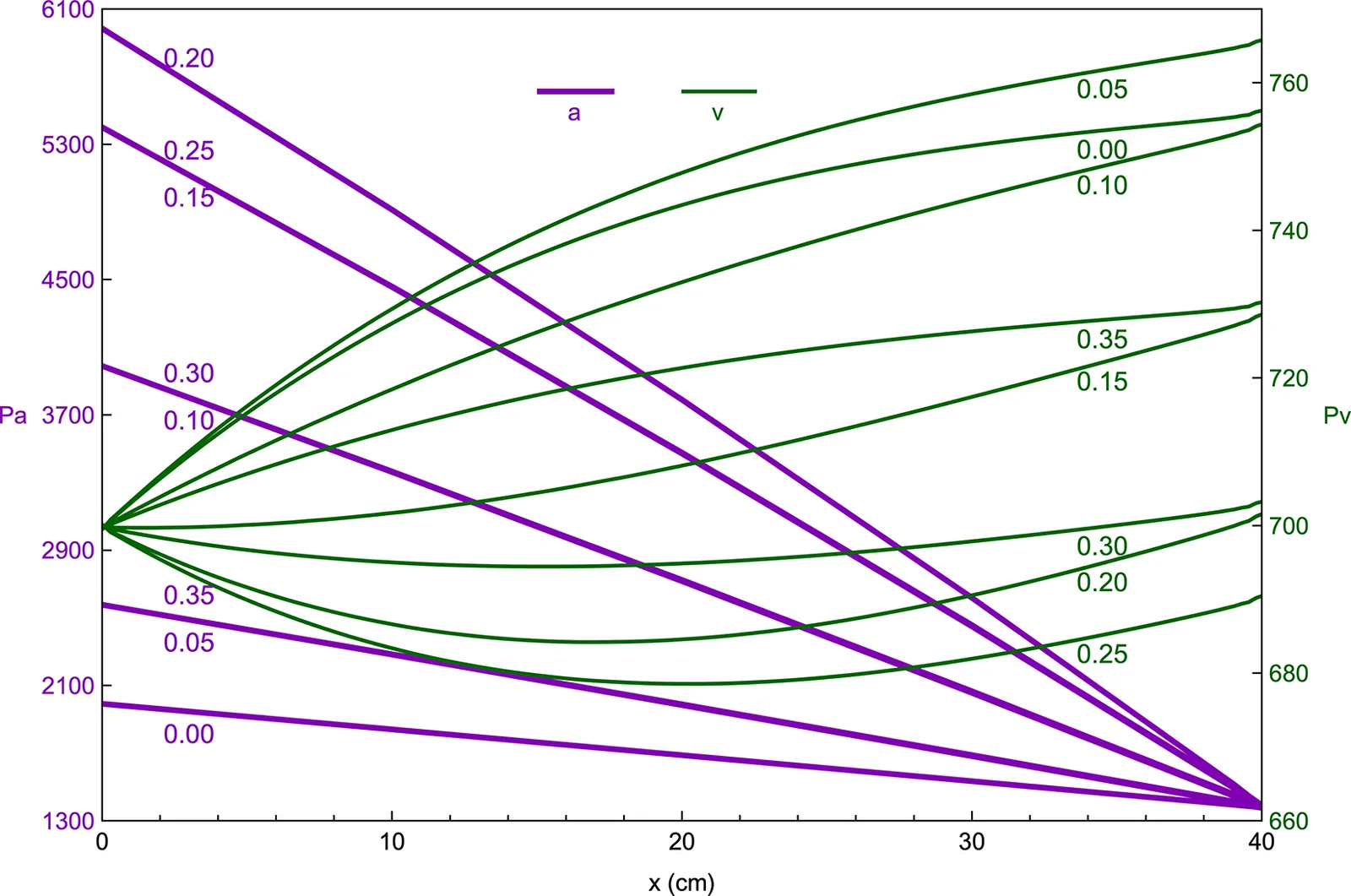
Space-time evolution of the pressure in the arteries ($P_{a},\;$thick lines and values on the left axis) and in the vein ($P_{v}$, thin lines and values on the right axis) for the reference case (parameters given in Table 1). The horizontal axis is the position x [cm] from the fetus. The labels on the different curves indicate the time in s of the represented snapshots of the evolution.

Figs 4 and 5 help to describe the behaviour in more detail (the numerical data associated with Figs 4-5 are provided in the S1 File). The different panels of Fig 4 are time plots of several important quantities in the system, with the time interval limited to 0.45 s (slightly more than 1 heartbeat). In Fig 4a, the time evolution of the pulsating (axial) flow rates in the different parts of the system is described. The flow rate in the arteries, denoted $Q_{a}(t)$, is represented for the central part of the cord (x=20 cm), but note the flow rate at other x-values is in fact the same and is therefore not represented. The other three (green) curves describe the flow rate $Q_{v}(t)$ in the vein at 10, 20, and 30 cm from the fetus side of the cord. Note that the different plotted flow rates are non-dimensional, with the mean flow rate $\overset{―}{Q}$ used as the scale. This quantity is of course a very important physiological parameter since it provides a metric for the exchanges between the mother and the fetus and its value in the reference case is$\;\overset{―}{Q}=\text{634 }\mu \text{l }{\text{s}}^{-\text{1}}$. In Fig 4b, the time evolution of the areas of the cross sections of the vein and arteries in three slices perpendicular to the cord axis are represented. The slices are located respectively at 10, 20 and 30 cm from the fetus and the plotted surfaces are normalized with respect to their time-averaged values. For further discussion, note that a detailed analysis of the numerical data shows that the time-averaged value of the vein cross-section area decreases by about 2‰ between x=30 cm and x=10 cm and can therefore be considered as almost constant along the vein. Fig 4c describes the time evolution of the pressure field in the vein, arteries and placenta. For the vein and arteries, the plotted quantity is the spatially averaged pressure in cross-sections x= constant. Note also that the different curves correspond to (P− $\overset{―}{P})/(\Delta P{)}_{max}$, where $\overset{―}{P}$ is the time averaged pressure and $(\Delta P{)}_{max}$ the amplitude of the pressure variations. Finally, Fig 4d describes the time evolution of the pressure gradient dP/dx in the vein at the same locations for which the pressure was plotted in Fig 4c. Fig 5 consists of a space-time description of the pressure field in the umbilical cord and provides therefore a description of the pressure wave in the system. The horizontal axis of the figure is the x-position along the cord, while the different curves correspond different instants. Both the pressure waves in the vein ($P_{v}$, thin curves) and in the arteries ($P_{a}$, thick curves) are represented, using left and right vertical pressure axes. Note that in the arteries, the plotted quantity is the spatially averaged pressure in cross-sections x= constant, while in the vein, the plot describes the pressure on the axis of the cord.

**Fig 6:**
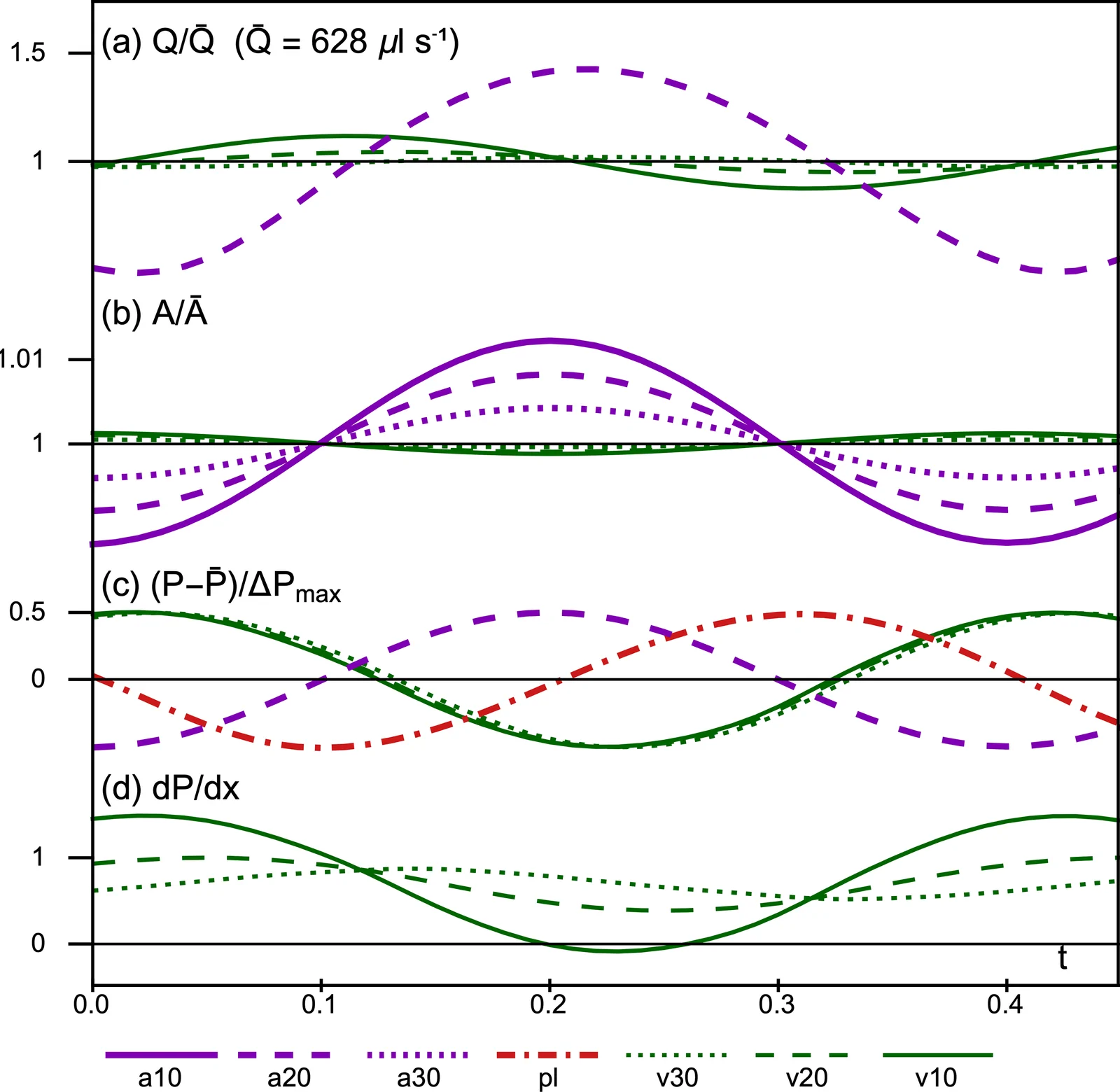
Time evolution of important quantities in the system when the Young moduli of the vessels’ walls and of WJ are doubled with respect to the reference case and with other parameters as in the reference case (see Table 1; plots slightly smoothened in order to remove irrelevant numerical noise). In the labels of the curves, *a*, *v* and *pl* refer to the arteries, the vein and the placenta respectively and the accompanying numbers (a30, or v10, for instance) indicate the position x along the cord, measured from the fetus and expressed in cm, for which the different quantities are plotted. (a) Relative flow rates $Q/\overset{―}{Q}$, where $\overset{―}{Q}$ is the time average of the flow across the cord. (b) Relative areas of the cross-sections $A/\overset{―}{A}$ for the two arteries and for the vein, with $\overset{―}{A}$ is the time average of these areas. (c) Pressures expressed in terms of the time averaged pressures $\overset{―}{P}$ and the amplitudes of the pressure variations $(\Delta P{)}_{max}$. (d) Pressure gradients (note that x is expressed in cm).

**Fig 7:**
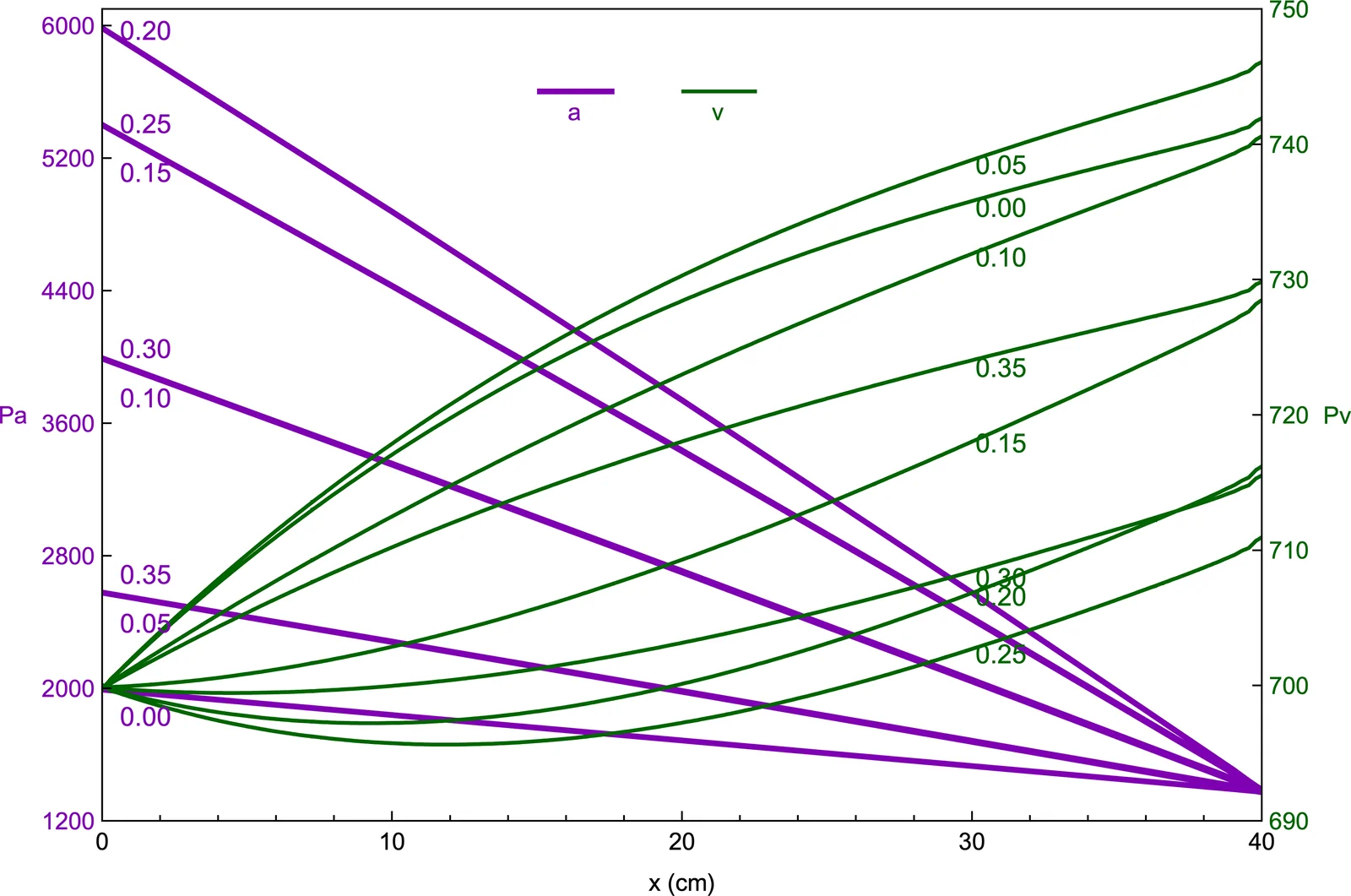
Space-time evolution of the pressure in the arteries ($P_{a},\;$thick lines and values on the left axis) and in the vein ($P_{v}$, thin lines and values on the right axis) when the Young moduli of the vessels’ walls and of WJ are doubled with respect to the reference case and with other parameters as in the reference case (see Table 1). The horizontal axis is the position x [cm] from the fetus. The labels on the different curves indicate the time in s of the represented snapshots of the evolution.

The results described by Figs 4-5 will be analysed and discussed in detail in Section 4.1. To prepare this discussion and the associated physical interpretations, it is important to present here additional results corresponding modified values of some important parameters of our system. Since the coupling of the flows in the arteries and in the vein is mediated by the deformations and displacements of the vessels’ walls and of WJ, we will first asses the influence of this coupling on the flows and on the general behaviour of the system by changing the elastic properties of both the vessels’ walls and of WJ. Figs 6-7 (the numerical data associated with Figs 6-7 are provided in the S2 File) provide the same plots as those given in Figs 4-5, but the Young’s moduli $E_{a}$, $E_{v}$, $E_{WJ}$ have been increased by a factor of 2 (all the other parameters being unchanged).

**Fig 8:**
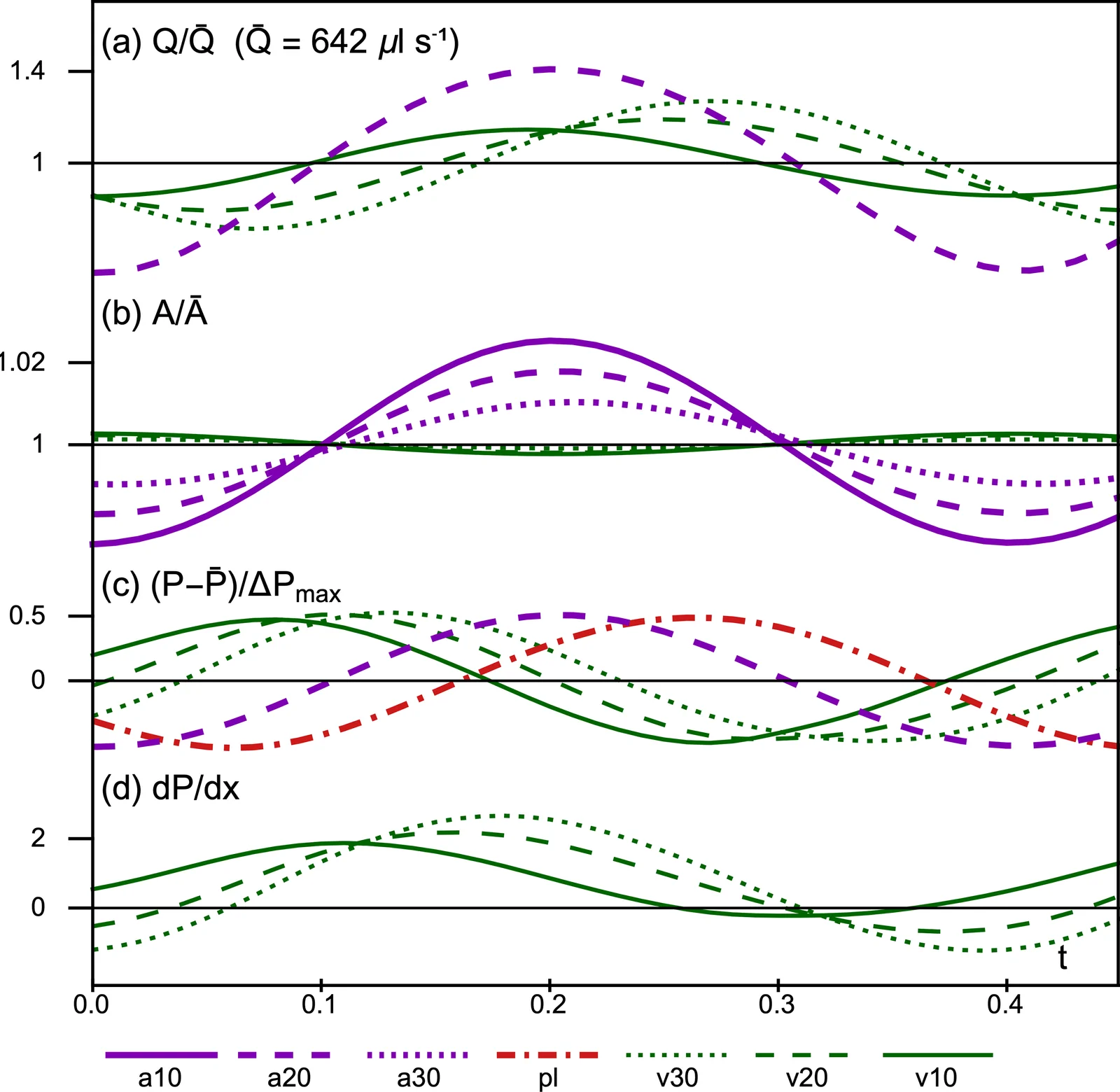
Time evolution of important quantities in the system when the compliance of the placenta is reduced to 2.5% of the value corresponding to the reference case and with other parameters as in the reference case (see Table 1; plots slightly smoothened in order to remove irrelevant numerical noise). In the labels of the curves, *a*, *v* and *pl* refer to the arteries, the vein and the placenta respectively and the accompanying numbers (a30, or v10, for instance) indicate the position x along the cord, measured from the fetus and expressed in cm, for which the different quantities are plotted. (a) Relative flow rates $Q/\overset{―}{Q}$, where $\overset{―}{Q}$ is the time average of the flow across the cord. (b) Relative areas of the cross-sections $A/\overset{―}{A}$ for the two arteries and for the vein, with $\overset{―}{A}$ is the time average of these areas. (c) Pressures expressed in terms of the time averaged pressures $\overset{―}{P}$ and the amplitudes of the pressure variations $(\Delta P{)}_{max}$. (d) Pressure gradients (note that x is expressed in cm).

**Fig 9:**
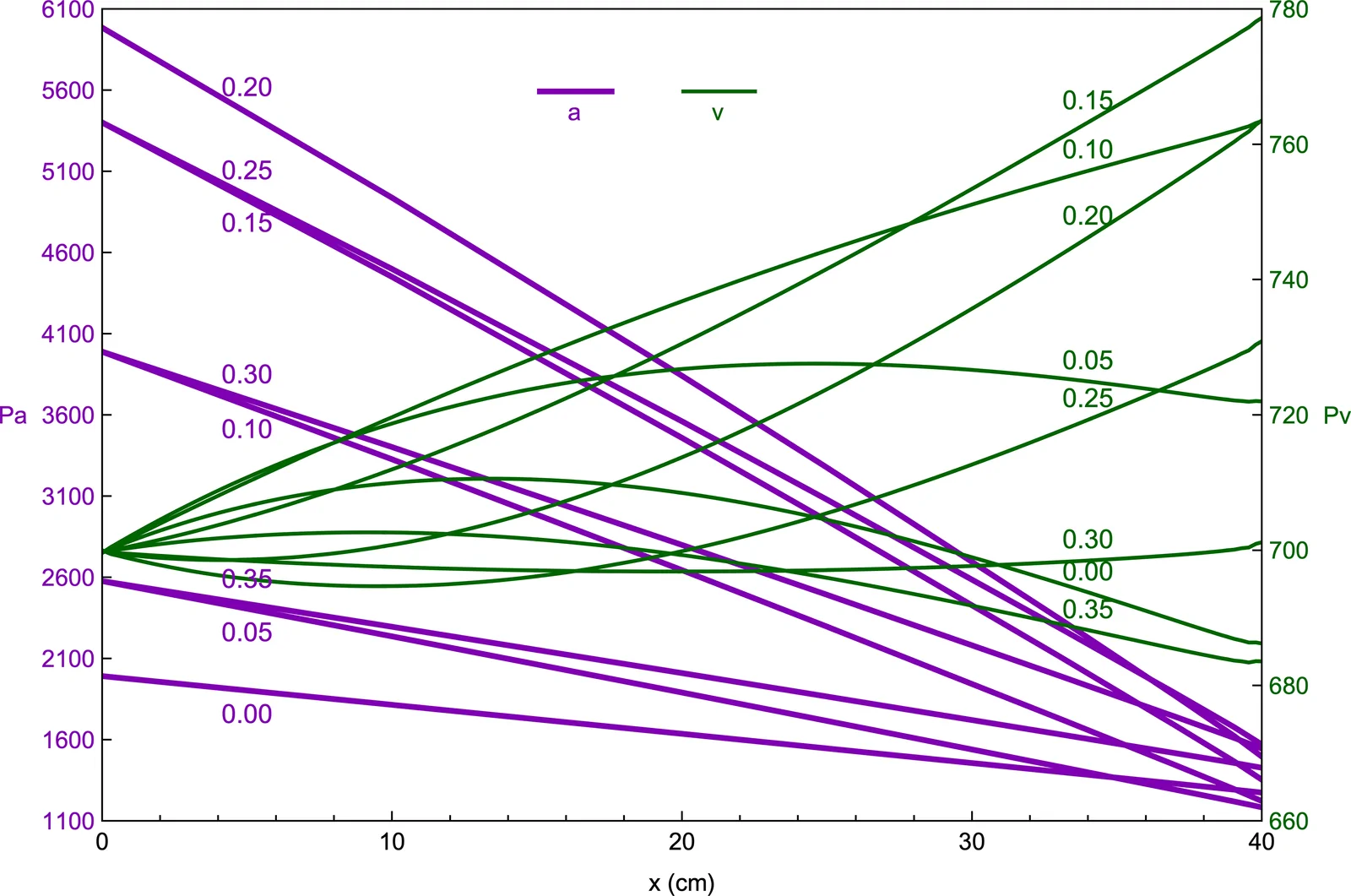
Space-time evolution of the pressure in the arteries ($P_{a},\;$thick lines and values on the left axis) and in the vein ($P_{v}$, thin lines and values on the right axis) when the compliance of the placenta is reduced to 2.5% of the value corresponding to the reference case and with other parameters as in the reference case (see Table 1). The horizontal axis is the position x [cm] from the fetus. The labels on the different curves indicate the time in s of the represented snapshots of the evolution.

Then, to assess the influence of the placenta on the behaviour of the system, and in particular its role in phase-shifting the flow between the arteries and the vein, we have considered a modified compliance $C_{p}$ for the placenta. In Figs 8-9 (the numerical data associated with Figs 8-9 are provided in the S3 File), we provide again the same plots as in Figs 4-5, but the compliance of the placenta is now $C_{p}=\text{0}.\text{1}\times \;{\text{1}\text{0}}^{-\text{9}}{\text{m}}^{\text{3}}/\text{Pa}$ (all the other parameters being unchanged). That value is 40 times smaller than in the reference case, which makes the placenta much less compliant, or much more rigid than in Figs 4-5.

### 3.2 Experimental results

**Fig 10:**
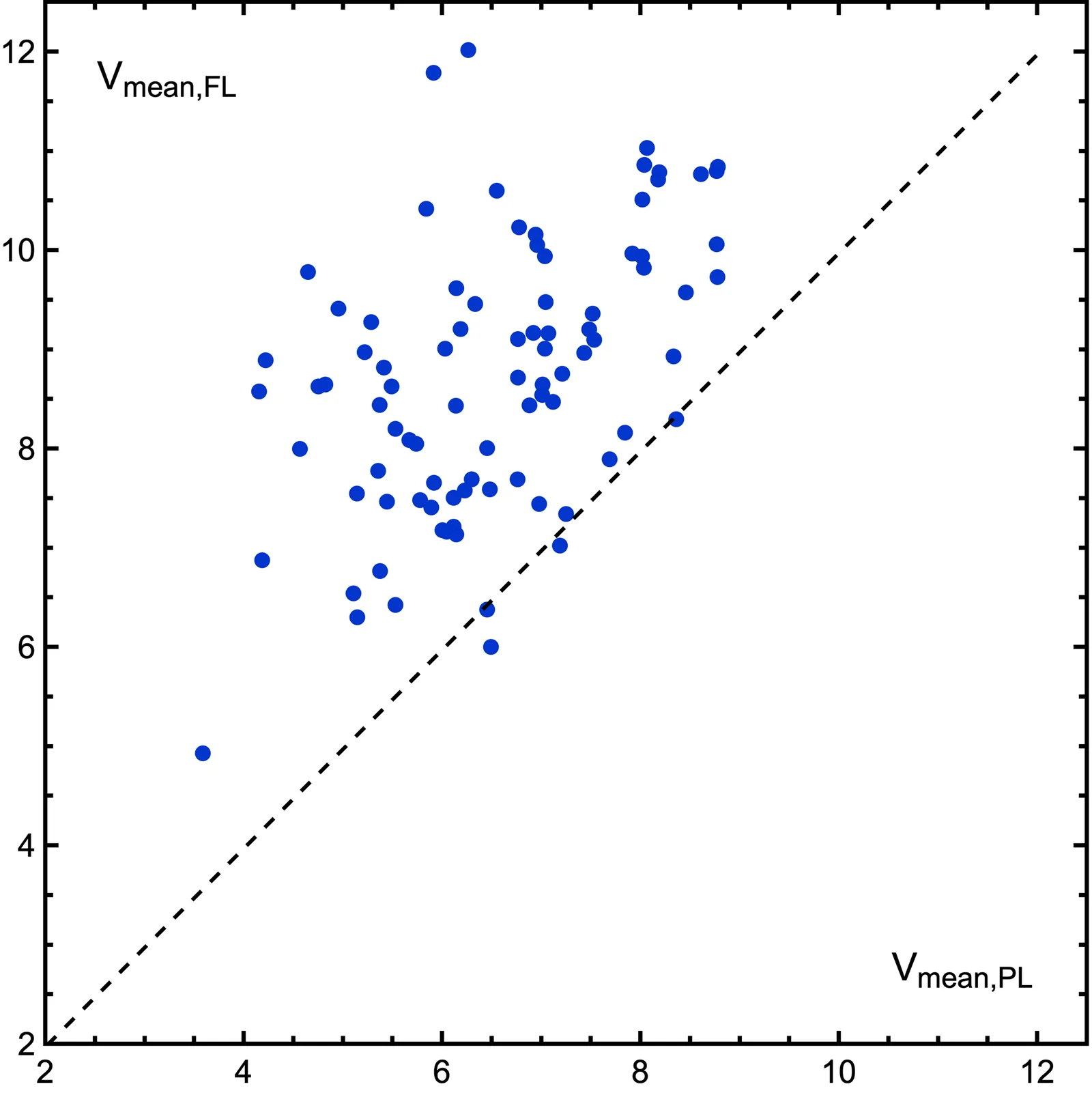
Comparison between the mean umbilical venous blood velocity (cm/s) measured at the placental insertion ($V_{mean,PL}$) and in a free-floating loop ($V_{mean,FL}$) of the umbilical cord. Each dot represents an individual fetus. The dashed line corresponds to the identity line and consistently highlights higher venous velocities in the floating loop than at the placental insertion site. Refer to Table 2 for statistics.

**Fig 11:**
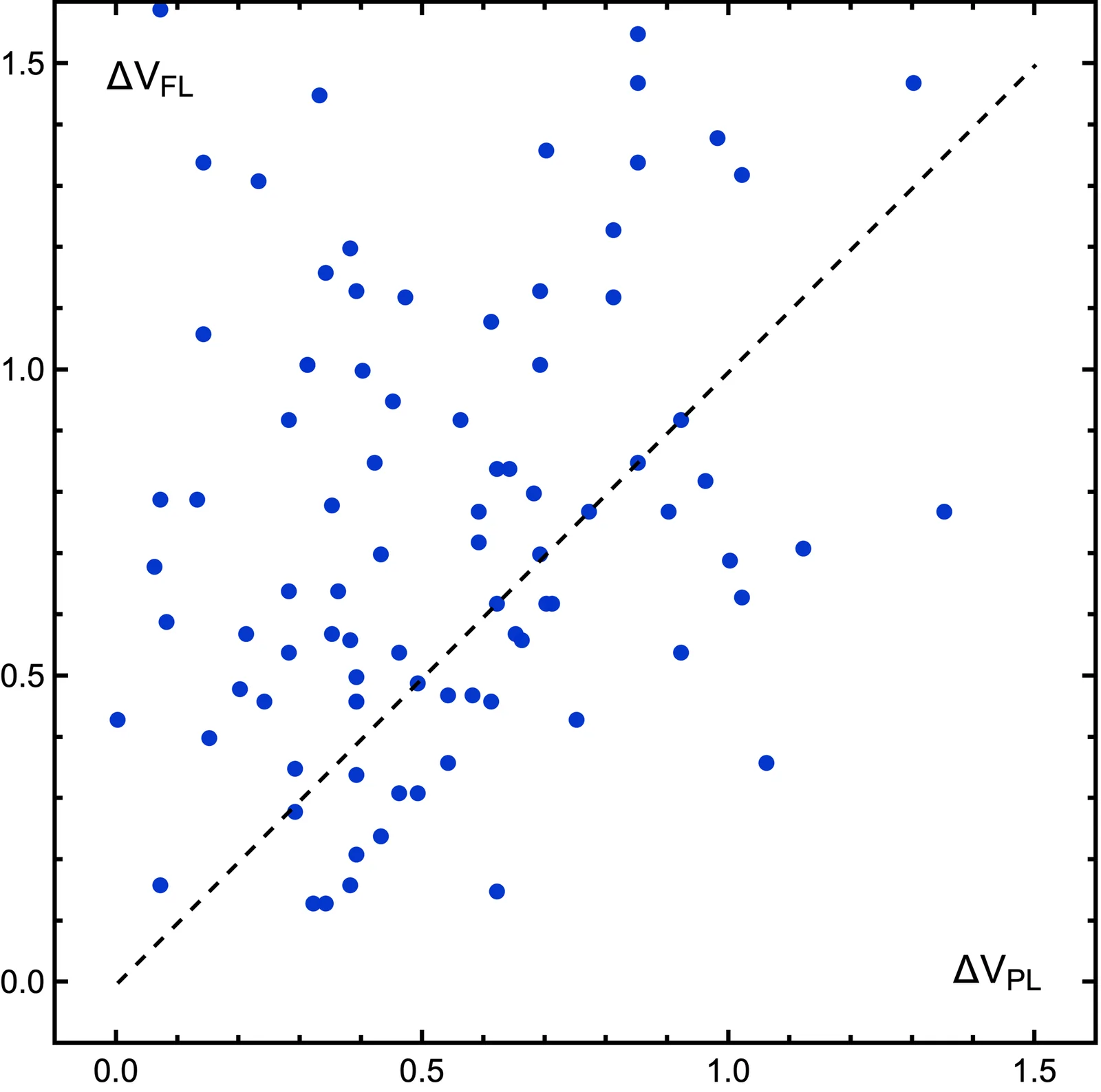
Comparison of umbilical venous velocity oscillation amplitude (cm/s) at the placental insertion ($\Delta V_{PL}$) and in a free-floating loop ($\Delta V_{FL}$) of the umbilical cord. Each dot represents an individual fetus. The dashed line corresponds to the identity line and consistently highlights higher velocity oscillation amplitudes in the floating loop than at the placental insertion site. Refer to Table 2 for statistics.

Our experimental results are presented in Figs 10 and 11 and the associated statistics are summarized in Table 2 (the experimental data associated with Figs 10-11 are provided in the S4 File).

**Table 2:** Statistics of the experimental results.

Variable [units]	N	Mean	SD	Min	Max
Gestational age [days]	88	88.4	4.23	79.0	98.0
$V_{S,PL}\;[$cm/s]	88	6.77	1.24	3.92	8.95
$V_{D,PL\;}$ [cm/s]	88	6.25	1.22	3.23	8.62
$V_{S,FL}$ [cm/s]	88	9.10	1.38	5.51	12.3
$V_{D,FL}\;[$cm/s]	88	8.35	1.41	4.38	11.7
$\Delta V_{PL}\;[$cm/s]	88	0.53	0.30	0.00	1.35
$\Delta V_{FL}$ [cm/s]	88	0.75	0.37	0.13	1.59
$V_{mean,PL}\;[$cm/s]	88	6.51	1.22	3.58	8.77
$V_{mean,FL}$ [cm/s]	88	8.72	1.38	4.95	12.0

The gestational age at the time of ultrasound evaluation was 12 weeks and 4 days ±4.23 days. The mean umbilical venous blood velocity over a beat, $V_{mean}$, measured in the free-floating loop of the cord was significantly higher than that measured near the placental insertion, with $V_{mean,FL}=\text{8}.\text{7}\text{2}\;\pm \;\text{1}.\text{3}\text{8}\text{ cm}/\text{s}$, $V_{mean,PL}=\text{6}.\text{51}\pm \;\text{1}.\text{22 cm}/\text{s}$ and a mean paired difference equal to 2.21 cm/s ± 1.31 cm/s, corresponding to a 34% increase (p<0.001) (see Fig 10). No difference has been noted in terms of umbilical vein diameters, with $D_{FL}=\text{1},\text{2}\text{9}\;\pm \;\text{0},\text{2}\text{2}\text{ mm}$, $D_{PL}=\text{1},\text{3}\text{0}\;\pm \;\text{0},\text{2}\text{4}\text{ mm}$. Note also that the evaluation of the ICC for velocity blood flow measurements in the umbilical vein showed a satisfactory agreement at both measurement sites, with ${ICC}_{FL}=\text{0}.\text{8}\text{4}\;(\text{95}\%\text{ CI}:\;\text{0}.\text{45}-\text{0}.\text{95})$ and ${ICC}_{PL}=\text{0}.\text{7}\text{9}\;(\text{95}\%\text{ CI}:\;\text{0}.\text{50}-\text{0}.\text{93})$. Finally, we have also observed that the amplitude ΔV of velocity oscillations in the vein was significantly greater in the free-floating loop of the umbilical cord than at the placental insertion site of the umbilical vein, with ${\Delta V}_{FL}=\text{0}.\text{7}\text{5}\;\pm \;\text{0}.\text{3}\text{7}\text{ cm}/\text{s}$, ${\Delta V}_{PL}=\text{0}.\text{5}\text{3}\;\pm \;\text{0}.\text{3}\text{0}\text{ cm}/\text{s}$.and a mean difference equal to 0.22 ± 0.42 cm/s (p<0.0001) (see Fig 11).

## 4 Discussion

### 4.1 Numerical results

We discuss now the results corresponding to the reference case. Fig 4b emphasizes first that the arteries cross-section has a minimum surface at t=0, which corresponds to the minimum pressure in the arteries. Similarly, the maximum expansion of the arteries corresponds to the maximum pressure. These results emphasize that the imposed pressure at the entrance of the arteries is directly responsible for the compression and expansion of these vessels. It is also interesting to note that the three curves describing the arteries expansion and contraction are in phase with one another, and also in phase with respect to the imposed pressure variations at the fetus side of the cord. This is a consequence of the very large pulse wave velocity in the arteries, whose walls are rather rigid. The plots also show that the amplitude of the arteries compression and expansion decreases when approaching the placenta, which is a consequence of the pressure decrease along the flow in these vessels. Let us now analyse the expansion and compression of the vein. Fig 4b shows that the three curves associated to the vein are phase-shifted by π with respect to those corresponding to the arteries. Moreover, the amplitude of the variations decreases when approaching the placenta, as in the arteries, even if the flow is in the opposite direction. This emphasize that the deformation of the vein is mechanically and directly induced by those in the arteries, while the pressure wave in the vein, and the pressure decrease along the flow seem to have almost no influence as far as the compression-expansion in the vein is concerned (see also below the discussion on pressure). It is then worth noting that the amplitudes of surface variations are much larger in the arteries than in the vein, which could seem surprising since total volume of the cord is unchanged. This can be explained by the fact that the plots describe relative variations of the surfaces (with the cross-section of the vein larger than that associated with the two arteries), and also by the compressibility of WJ, which can therefore absorb part of the deformations imposed by the arteries.

The blood pressure evolution in the vessels is described by Fig 4c, which allows observing the phase-shifts between the pressure waves measured at different places in the system, and by Fig 5, which consists of a description of the pressure wave in the system. The pressure wave is generated by the imposed pressure at the arteries entrance. Its propagation in the arteries towards the placenta and back to the fetus in the vein is a complex phenomenon involving many physical effects which interact in an intricate way. First, it is well known that the elasticity of the vessels’ walls allows the propagation of perturbations, with reflection phenomena at the different boundaries of the system [56]. The compliant placenta dampens and delays the wave. The presence of the deformable Wharton’s jelly also permits some mechanical coupling between the arteries and the vein. The flow inside the vessels has some additional influence on the propagation of pressure waves, while non-linearities and inertia have also an important role, especially in the case of pulsatile flows [57–59]. Our numerical model accounts for all these effects and the calculated behaviour is therefore the result of the complicated interactions between all these physical mechanisms. Fig 5 describes the pressure wave in the vein and arteries. In the arteries, the pressure decrease along the flow is linear at each instant but note that in a very small part of the system close to the placenta (~0.395<x<0.4,  not visible at the scale of the picture), the behaviour is a bit more complex due to the influence of the compliant placenta. Let us also mention that the mean pressure at the arteries’ ends, which corresponds in our model to the pressure in the placenta, is 1385 Pa, while the amplitude of the wave is around 10 Pa, which is very small because of the high compliance of the placenta which absorbs and dampens the wave. In the vein, the x- dependence of the pressure is not linear, at least partly due to the reflection phenomena induced by a fixed pressure at the exit. It is also worth noting that the amplitude of the wave at the entrance of the vein is around 75 Pa, which is a larger value than at the exit of the arteries. The plot (or the associated data) also shows that the amplitude of the wave decreases along the flow (*i.e.,* towards the fetus), while we have seen above that the amplitude of the surface variations decreases when approaching the placenta. This surprising result emphasizes the complicated interactions of the various mechanisms at work in the system. Fig 4c allows analysing phase-shifts between the pressure waves considered at several locations in the system (10, 20 and 30 cm from the fetus). To simplify the analysis, the curves have been rescaled in such a way that for each location, the plot represents the deviation from the mean value, this deviation being measured as a fraction of the amplitude of the variations ($\Delta {P)}_{max}$. For arteries, a unique curve, corresponding to the central part of the cord (x=20), is provided because the curves corresponding to other places would be the same (except for curves very close to the placenta, for ~0.395<x<0.4 ). The curve describing the placenta shows a phase lag of about π/2 with respect to the one for arteries, while the three curves for the vein, which are almost perfectly in phase, show a phase lag slightly larger than π with respect to the one for arteries. Consider now the pressure gradient dP/dx in the vein, which is represented in Fig 4d, and which can also be analysed qualitatively by observing the slopes of the (thin) curves of Fig 5. The amplitude of variations of dP/dx increases when approaching the fetus and close to the fetus (x=10 cm), the pressure gradient becomes negative during part of the evolution. Note also that the three curves of Fig 4d are not in phase, even if the pressure waves in Fig 4c were so. These results will be important in analysing the flow in the vein below.

Consider now the evolution of the blood flow rate, as described by Fig 4a. The first minimum of the flow rate $Q_{a}(t)$ in the arteries appears at a small value of t (t≈0.02 s), which means that $Q_{a}(t)$ lags slightly behind the imposed entrance pressure, the latter being also in phase with the deformation of the vessel. Obviously, this small delay originates in the inertia of the blood. The three curves $Q_{v}(t)$ are phase-shifted with respect to $Q_{a}(t)$, and also with respect to one another. The origin of these phase-shifts is associated with several physical effects, already alluded to above (compliance of the placenta and of the vessels, reflection of wave, mechanical interactions through WJ, non-linearities). The respective contributions of these effects to the behaviour will not be analysed further, but it is interesting to compare the different curves $Q_{v}(t)$ with the pressure gradient curves of Fig 4d. First, we can observe that the amplitude of the time variations of both $Q_{v}$ and dP/dx changes along the vein (different values at x=10, 20, or 30 cm) and increases when approaching the fetus (larger amplitude for the full curves than for the dashed and dotted lines). One can also notice that in Figs 4a and 4d, the three plotted curves show similar phase-shift with respect to one another and that the pressure gradient curve is always slightly in advance with respect to the corresponding flow rate. All these remarks emphasize a close connection between the pressure gradient and the flow, the former being induced by the complex propagation of the pressure wave induced at the entrance of the arteries, and also providing a kind of driving force for the latter. A sort of oscillatory Poiseuille flow is created, in which the blood inertia induces the phase lag of $Q_{v}$ with respect to dP/dx. As a final remark, note that in a rigid cord, *i.e.,* in the absence of arteries-vein interactions, the amplitude of the variations of the flow rate along the vein would of course be constant with respect to x. The increase of this amplitude when approaching the fetus, combined with a decrease of the amplitude of the pressure variations (see Fig 5), is thus a slightly surprising feature of the behaviour of the system and will be discussed further below, notably in connection with experiments (see Section 4.2).

After analysing the reference case, we consider now increased Young’s moduli $E_{a}$, $E_{v}$, $E_{WJ}$ (reduced deformations and mechanical interactions between arteries and vein) and a reduced compliance for the placenta (more rigid placenta). Figs 6-7 correspond to increased Young’s moduli and comparison of Figs 6b and 4b directly shows the reduction of the deformations in the arteries and vein. Regarding the phase-shifts of the pressure wave considered at different places in the system, Figs 6c and 7 do not feature significant changes with respect to what was observed in the reference case. Fig 7 also shows that the amplitude of the pressure wave at the entrance of the vein is now around 35 Pa, and thus notably reduced with respect to the reference case (75 Pa). This parameter is thus directly influenced by the arteries-vein interactions. The pressure gradient presented in Fig 6d does not either feature important qualitative changes with respect to the reference case but note however that the time window with negative values and the amplitude of the variations are reduced with respect to Fig 4d. It is then worth noting that the mean blood flow rate is reduced in a more rigid cord, with $\overset{―}{Q}=\text{628 }\mu \text{l }{\text{s}}^{-\text{1}}$, instead of $\text{634 }\mu \text{l }{\text{s}}^{-\text{1}}$ in the reference case, which corresponds to a flow rate reduction of about 1%. Even if the numerical value of the change is rather small, this blood flow rate reduction in a more rigid cord is a very important result because it emphasizes that the interactions between the arteries and the vein actually promote efficient blood circulation in the cord and therefore increase the exchanges between the mother and the fetus. This also provides a numerical proof for the existence of a pulsometer-like mechanism in the behaviour of the cord. Finally, note that the increase of the amplitude of the time variations of the flow rate when approaching the fetus, combined with a decrease of the amplitude of the pressure variations, is still present here, as in the reference case, but the effect is less important, which highlights, or confirms the role of the arteries-vein interactions in that feature of the behaviour.

The influence of a more rigid placenta is described by Figs 8-9. The time evolution of the area of the cross-sections of the arteries and vein is described in Fig 8b, which shows that the behaviour remains in fact qualitatively similar to the corresponding figure for the reference case (see Fig 4b), with the vein behaviour phase-shifted by about π with respect to the arteries. Note however that a very slight phase-shift is observed between the three curves describing the vein or between those corresponding to the arteries. The pressure wave is illustrated in Figs 8c and 9, which confirm a behaviour completely different from the reference case, with clearly visible phase-shifts along the vein and along the arteries (in arteries, the phase-shift becomes important for ~0.3<x<0.4, see Fig 9). Note also the large amplitude of the pressure wave at the end of the arteries, *i.e.,* in the placenta, which is around 391 Pa (10 Pa in the reference case). This result is a consequence of the large value of $C_{p}$, which characterizes a “rigid” placenta that does not dampen the wave as in the reference case. The amplitude of the wave at the vein entrance is around 95 Pa and is thus reduced with respect to the value in the placenta, which provides another important difference with respect to the reference case and shows that the arteries-vein interactions are strongly influenced by the placenta. The vein pressure-gradient curves presented in Fig 8d are also markedly different from those shown in Fig 4d. In particular, the three curves exhibit different phase relationships and, more importantly, the amplitude of the variations is larger at x = 30 cm than at x = 10 cm, which is the opposite of what is observed in the reference case. Finally, let us mention that the mean flow rate corresponding to $C_{p}=\text{0}.\text{1}\times \;{\text{1}\text{0}}^{-\text{9}}{\text{m}}^{\text{3}}/\text{Pa}$ is $\overset{―}{Q}=\text{642 }\mu \text{l }{\text{s}}^{-\text{1}}$, which is a larger value than in the reference case. Note also that the phase-shifts between the curves describing the flow rates in Fig 8a are completely changed with respect to Fig 4a. Moreover, the surprising increase of the amplitude of variation of the flow rate when approaching the fetus (with a still decreasing amplitude of the pressure variations, see Fig 9) has disappeared in the present situation, which confirms again the important role of the placenta in shaping the vein-arteries interactions, the phase-shifts along the system and the general behaviour of the umbilical cord.

### 4.2 Experimental results

Based on sonographic Doppler studies, we measured a 34% increase in $V_{mean}$ in the first segment of the umbilical vein, close to its placental insertion. Additionally, we confirmed a satisfactory inter-observer agreement for $V_{mean,FL}$ and $V_{mean,PL}$ (${ICC}_{FL}=\text{0}.\text{8}\text{4}\;$ (95% CI: 0.45−0.95) and ${ICC}_{PL}=\text{0}.\text{7}\text{9}\;(\text{95}\%\text{ CI}:\;\text{0}.\text{50}-\text{0}.\text{93}))$. In contrast, we did not observe any change in the vein diameter between the 2 measurement sites. Assuming that the venous flow can reasonably be considered as an oscillatory Poiseuille flow, in each cross-section, the instantaneous flow rate can be evaluated as half the product of the maximum velocity in that cross-section and the cross-section area. As far as the mean flow rate is concerned, the change in the mean velocity $V_{mean}$ between the two measurement sites, associated with an absence of change in the diameter, could seem surprising and should be interpreted in terms of the limitations of the present imaging approach, for which the geometric changes are too small to be detectable. Indeed, the variations are likely to remain below the spatial resolution of the ultrasound system used, particularly when inner-to-inner measurements are performed manually on static frames [60]. Moreover, Doppler-derived velocities may be influenced by changes in the spatial velocity profile along the cord: Pennati *et al*. [38] reported a transition from a flatter profile near the placental end to a more parabolic profile distally, which could contribute to higher peak and mean velocities without any change in diameter. So the measured increase in velocity $V_{mean}$ could be explained, at least partially, by diameter changes along the vein, but these geometrical variations are outside the resolution of our ultrasound system. The increase can also result from the modification of the velocity profile along the vein.

Another important result of our experimental approach is the observed change of the amplitude of the velocity oscillations along the vein. While umbilical venous flow is typically described as non-pulsatile in late gestation, subtle pulsatility can be physiological in early pregnancy [61]. Such pulsatility has actually been detected in our measurements and an important observation is the increase of the amplitude of these oscillations from the placenta towards the fetus, with ${\Delta V}_{FL}>{\Delta V}_{PL}$.

### 4.3 Comparison between our experimental and numerical findings and discussion in light of other works

In the cohort considered in our experiments, we have observed that the time average of the maximum spatial velocity in the cross-section of the vein increases from the placenta towards the fetus. Assuming an oscillatory Poiseuille-like flow, conservation of the (mean) blood flow rate then imposes a decrease of the cross-section area, but we have indicated that the limited accuracy of the measurement of the vein diameter prevented us from observing this geometrical effect in our experimental approach. On the other hand, the diameter of the vein in the reference configuration of our numerical model was assumed constant along the cord and the results of the calculations have also shown that the deformation induced by the flow are small and that the time-averaged vein cross-section area hardly changes along the cord. For that reason, a comparison between experiments and the numerical approach is not really possible for this effect.

In contrast, an interesting comparison is possible as far as the amplitude of the flow rate, or of the fluid velocity is concerned. The numerical model demonstrates that umbilical venous flow dynamics results from a complex interaction between arterial pulsatility, cord mechanical properties, and placental compliance. Depending on the relative stiffness of the cord components and the downstream placental impedance, venous velocity oscillations may be partially transmitted, damped, or locally amplified along the cord. Importantly, the model predicts that variations in venous flow rates amplitude can occur without necessarily inducing large changes in venous diameter. Moreover, for the so-called reference case (parameters in Table 1, results discussed in the beginning of Section 4.1), we have emphasized an increase of the amplitude of the flow rate variations from the placenta towards the fetus. Assuming an oscillatory Poiseuille-like flow, and because the vein diameter remains almost constant over time (see Fig 4c), this behaviour corresponds to an increase of the amplitude of the velocity oscillations ΔV, which is precisely what was observed in experiments. This agreement between experiments and the numerical results is a great achievement and provides an important validation of the numerical model. This finding also supports the interpretation that the observed increased ΔV in the free loop with respect to the vicinity of the placenta (${\Delta V}_{FL}>{\Delta V}_{PL}$) is a consequence of the intricate interactions between the arteries, the vein and the placenta (see discussion in Section 4.1) and reflect a redistribution of the pulsatile energy rather than a simple geometrical effect. Taken together, the concordance between numerical and *in vivo* data supports the concept that umbilical venous flow in early pregnancy is not strictly steady, but exhibits low-amplitude, site-dependent pulsatility shaped by the mechanical and hemodynamic properties of the feto-placental unit.

As in Reynolds’ work [1], we observe pressure variations in the umbilical arteries and vein that are in phase opposition. Reynolds reported this phenomenon based on measurements performed in the ovine fetus and proposed the hypothesis of a “pulsometer” mechanism, whereby iterative decreases in venous pressure, occurring synchronously with arterial pulsations, would be induced by periodic stretching of the venous wall. In contrast, our numerical model suggests a different mechanistic interpretation. Specifically, it indicates that the phase opposition between arterial and venous pressures arises from a complex interplay between arterial pulsatility, the mechanical properties of the umbilical cord, and placental compliance, rather than from a direct pulsatile suction effect on the vein. Furthermore, important anatomical and physiological differences must be considered when comparing these interpretations. The ovine umbilical cord differs structurally, with two arteries and two veins showing little or no coiling, unlike the human cord. In addition, placental architecture differs fundamentally between species: cotyledonary and epitheliochorial in sheep, versus discoid and hemochorial in humans [62]. This system-level interpretation is consistent with previous work emphasizing the mechanical role of WJ. In particular, Brunelli *et al*. proposed in [14] that the central core of WJ facilitates the transmission of arterial pulsations to the vein, generating pressure variations and contributing to venous return, with a possible gradient of mechanical properties along the cord. Although our model does not explicitly incorporate such a spatial gradient, our results show that increasing cord rigidity reduces both the amplitude of pressure variations and the mean venous flow. These findings support the idea that the mechanical environment of the cord, and potentially its spatial heterogeneity, plays a key role in optimizing fetoplacental hemodynamics.

### 4.4 Limitations

The system of interest in the present work is intricate and multifaceted. The material properties of the biological tissues that make up the umbilical cord are complex. Moreover, many interactions exist between the different parts of the system, and also with the outside of the umbilical cord and placenta system, both from the fetus and the mother side. A thorough description of all biophysical mechanisms at work is clearly out of reach and a tractable model of the system cannot be obtained without introducing important simplifications or approximations, which define the limitations of our work. Our most important assumptions or simplifications are the following. First, the material properties of the biological tissues and blood were not described in detail. Blood is a shear-thinning non-Newtonian fluid [63], whose properties depends also on age and temperature. However, these effects are not accounted for in our work, and we describe blood as an incompressible Newtonian fluid with constant viscosity. The tissues that make up WJ or the arteries’ and vein’s walls exhibit viscoelastic and hyperelastic behaviour and the associated material properties are reported in the literature with significant discrepancies among studies [41–50]. It is also important to stress that the values of the parameters *in vivo* are not precisely known because of an understandable lack of available mechanical characterisation of umbilical cord tissues during pregnancy. Most published data on material properties are derived from postnatal measurements, with no established consensus on how these properties evolve in utero. Because of these uncertainties on the true values of the parameters during pregnancy, we decided to use a linear Hookean model in our numerical calculations. The interactions and connections between the parts of the system and with the outside are also described in a simplified way. First, we have used a 0-D (or lumped-parameter model) for the placenta. Such 0-D or lumped-parameter models are common in hemodynamics (see [56] for instance) and have already been coupled to 3-D models as in [64,65]. In particular, it is worth noting that such a 0-D model is used for the placenta in [19,20]. A 1-D model, with spatially distributed compliance and resistance, or still more complex models [17,18] could also have been introduced. However, this would imply additional computational cost. Moreover, the 0-D formulation captures the dominant hemodynamic effects through equivalent localised resistance and compliance parameters, as well as wave reflection imposed by boundary conditions and thus contains the main ingredients of the connection between arteries and vein, whose 3-D interactions are the main subject of our analysis. Another hypothesis concerns the boundary conditions on the outer shell of the umbilical cord. Inside the womb, the cord can move and deform and assuming a rigid and fixed outer shell probably amplifies artificially the deformation that the pulsating arteries induce on the vein. However, the amniotic membrane surrounding the cord is rather relatively stiff [66,67] and the deformations of the cord are thus limited. Moreover, the bias induced by our boundary condition on the arteries-vein interactions is mainly quantitative, while the qualitative behaviour should not be called into question. The pressure conditions imposed at the arteries entrance and vein exit are also simplified descriptions of the situation. In particular, the imposed sinusoidal inlet pressure is not strictly realistic but allows describing the main frequency of the behaviour. This point will be discussed further in Section 5, where some possible future analyses are described. Finally, it is important to note one last limitation of our study: the discrepancy between the gestational age used in the experiments (11–14 weeks) and the one used in the numerical model (20–22 weeks). The discrepancy reflects in fact methodological constraints, which are different in experiments and in modelling. The first trimester offers more favourable technical conditions for Doppler measurements, while reliable and comprehensive hemodynamic and geometric data for the umbilical cord in early gestation remain scarce in the literature. For that reason, the parameters of our numerical model correspond to mid-gestation, which is a bit better documented.

In summary, it can be said that the limitations of our approach stem both from the complexity of the system, which cannot be described in every detail, and from the lack of available data that would allow us to accurately determine the values of the numerical model’s parameters. In this context, our work must be considered as a heuristic approach aimed at understanding a bit better the system’s behaviour, with a particularly focused interest on how the interactions between the arteries, the vein, and the placenta may influence blood flow in the cord and exchanges between the mother and the fetus. It must also be clear that our work does not claim to provide quantitative results, or quantitative agreement between experiments and modelling, but rather intend to emphasize qualitative tendencies in the system’s behaviour, which are hopefully not overly sensitive to the values chosen for the parameters of the model or the simplifications introduced.

## 5 Conclusion and future work

In this paper, a long-standing question about umbilical venous return mechanisms was studied by jointly investigating the mechanical and hemodynamic interactions between the umbilical arteries, the vein, and the placenta. To this end, a numerical fully coupled three-dimensional fluid–structure interaction model of the umbilical cord was developed and connected to a zero-dimensional model of a compliant placenta. In parallel, an experimental Doppler ultrasound study was conducted in early human pregnancy (11–14 weeks of gestation) to explore whether the mechanisms predicted numerically have measurable physiological correlates *in vivo*. By combining numerical simulations and experimental observations, this study aimed to provide a unified and mechanistic understanding of feto-placental blood flow regulation.

The numerical model demonstrates that umbilical venous flow is not governed solely by the imposed pressure gradient but emerges from a complex interplay between arterial pulsatility, umbilical cord mechanics, and placental compliance. First, arterial pressure oscillations induce cyclic expansions of the arteries, which are mechanically transmitted through Wharton’s jelly to the vein, leading to periodic venous deformation but phase-shifted relative to arterial motion. Second, despite the small amplitude of arterial pressure oscillations transmitted through the highly compliant placenta (≈10 Pa in the reference case), the resulting venous pressure gradients generate measurable oscillations in venous flow. Third, the simulations reveal an interesting increase in the amplitude of venous flow oscillations when approaching the fetus, even though the amplitude of venous pressure variations decreases along the same direction. This effect is reduced for more rigid cord and completely disappears for low placenta compliance, highlighting the essential role of both arterial–venous mechanical coupling and placental impedance. Finally, stiffening the cord components leads to a reduction of the mean flow rate, providing numerical evidence that arterial–venous interactions enhance overall venous return and supporting the existence of a pulsometer-like mechanism within the umbilical cord.

The experimental Doppler ultrasound study provides *in vivo* evidence that umbilical venous flow in early pregnancy is not strictly steady, as predicted by the numerical model. A significant increase in the time-averaged maximum venous velocity was observed between the placental insertion and the free-floating loop of the cord (+ 34%, p<0.001), while the accuracy of the measurement did not allow emphasizing a significant difference in vein diameter between the two sites. In addition, reproducible low-amplitude venous velocity oscillations were identified, with a significantly greater oscillation amplitude in the free-floating loop compared to the placental region. These findings indicate that venous pulsatility is a physiological feature of early gestation and that both mean velocity and pulsatile components of venous flow vary along the cord, despite the absence of measurable geometric changes using conventional ultrasound techniques.

The concordance between calculated predictions and experimental observations provides an important validation of the numerical approach and represents a key outcome of this study. The numerical model predicts a site-dependent oscillating venous flow rate, with an increasing amplitude along the cord towards the fetus, this behaviour being fully consistent with the Doppler findings. Moreover, the simulations provide a mechanistic explanation for the experimental observations, attributing it to the redistribution of pulsatile energy resulting from arterial pulsations modulated by cord elasticity and placental compliance. Together, the numerical and experimental approaches support a revised view of umbilical venous flow in early pregnancy as a dynamic, mechanically modulated process.

Our results provide additional insight into the fundamental mechanisms governing fetoplacental hemodynamics. By highlighting the role of the interaction between arterial pulsatility, cord mechanical properties, and placental compliance, our model suggests that optimal venous return depends not only on pressure gradients but also on the biomechanical environment of the cord–placenta unit. This has direct implications for artificial womb design. Current systems aim to reproduce the fetal circulation in a pumpless or low-resistance configuration, yet our findings suggest that reproducing physiological flow patterns may also require careful consideration of mechanical coupling and compliance within the system. In particular, failure to adequately mimic the distributed mechanical properties of the native fetoplacental unit may contribute to the hemodynamic instability and cardiac dysfunction observed in experimental models. Thus, beyond their physiological significance, our results may help inform the design and optimization of extracorporeal support systems, with the goal of preserving more physiological venous return and minimizing cardiovascular stress in extremely preterm fetuses.

Several limitations or shortcomings have been acknowledged above and the issues raised suggest various avenues for future modelling. First, a more realistic description of the behaviour of the blood could be introduced, considering non-Newtonian behaviour. Similarly, future work should aim to incorporate more realistic constitutive laws for the vessels’ walls and for Wharton’s jelly, or explore subject-specific geometries, but these improvements depend on the availability of data to determine the parameters. It would also be interesting to avoid the artificially imposed sinusoidal pressure difference at the fetus side of the cord by introducing a model of the fetus heart and an additional lumped-parameter model of the hemodynamics of the fetus [68–71]. A more detailed model of the placenta could also be considered. In particular, a 1-D model, with spatially distributed compliance and resistance, would provide a more realistic description of wave reflections and phase-shifts in the system. On the experimental side, high-frame-rate ultrasound or advanced imaging modalities could allow simultaneous measurement of velocity and diameter dynamics.

## Supporting information

S1 File S1.xlsx. The numerical data associated with Figs 4–5 are provided in the S1 file.
(XLSX)

S2 File S2.xlsx. The numerical data associated with Figs 6–7 are provided in the S2 file.
(XLSX)

S3 File S3.xlsx. The numerical data associated with Figs 8–9 are provided in the S3 file.
(XLSX)

S4 File S4.xlsx. The experimental data associated with Figs 10–11 are provided in the S4 file.
(XLSX)
